# Nanofiber Mats as Amine-Functionalized Heterogeneous Catalysts in Continuous Microfluidic Reactor Systems

**DOI:** 10.3390/gels12010055

**Published:** 2026-01-06

**Authors:** Katja Rumpke, Naresh Killi, Barbara Dittrich, Andreas Herrmann, Dirk Kuckling

**Affiliations:** 1 Department of Chemistry, Paderborn University, Warburger Str. 100, 33098 Paderborn, Germany; krumpke@mail.uni-paderborn.de (K.R.); naresh.killi@uni-paderborn.de (N.K.); 2 DWI—Leibniz-Institut für Interaktive Materialien e.V., Forckenbeckstr. 50, 52074 Aachen, Germany; dittrich@dwi.rwth-aachen.de (B.D.); herrmann@dwi.rwth-aachen.de (A.H.); 3 Institute for Technical and Macromolecular Chemistry, Rheinisch-Westfälische Technische Hochschule (RWTH) Aachen University, 52074 Aachen, Germany

**Keywords:** heterogeneous catalysis, flow chemistry, sustainable synthesis, organo-catalysis, nanomaterials, green chemistry

## Abstract

The development of sustainable catalysts is the main objective in green chemistry approaches. In this study, a catalytically active polymer based on a tertiary amine was synthesized, functionalized with a photo-crosslinker, and structured into nanofibers via electrospinning technique with polycaprolactone (PCL) as a stabilizing additive. Subsequent photo-crosslinking yielded hierarchically porous polymers with high swelling properties and increased surface areas, thereby improving the accessibility of the immobilized catalytically active sites. The nanofiber mats were incorporated into a microfluidic reactor (MFR) setup and utilized as heterogeneous catalysts for the Knoevenagel reaction of malononitrile with different aldehydes. It was observed that the system demonstrated a substantial improvement in NMR yields (40–60%) and turnover frequencies (50–80 h^−1^) in comparison to catalytical systems that had been previously published. Reusability studies showed reproducibility of NMR yields over up to three cycles. The obtained results demonstrate the potential of electrospun, photo-crosslinked nanofibers as efficient heterogeneous catalysts in microfluidic synthesis, thus contributing to more sustainable production of valuable malononitrile derivatives.

## 1. Introduction

In addition to the pursuit of scientific advancement, chemists play a pivotal role in shaping the trajectory of research and development towards sustainability and circularity, in the context of global environmental changes. In this manner, the guidelines embody the principles of green chemistry, encompassing the avoidance of waste, the utilization of safe solvents and conditions, the maximization of atom economy, the minimization of energy consumption, the facilitation of reuse, the prevention of harmful releases and the usage of catalysts [1,2]. One potential approach regarding this is the combination of microflow chemistry and heterogeneous catalysis. It is evident that the utilization of continuous flow devices confers numerous advantages over conventional batch processes. These advantages include a substantial surface-to-volume ratio, precise mixing, efficient mass and heat transfer, intrinsic safety, reduced solvent usage, scalability and enhanced turnover [3,4]. The implementation of additional catalysis facilitates the enhancement of atom economy through the optimization of turnover, while ensuring that energy input remains at a minimum. In this context, considerable attention has been drawn to the immobilization of homogeneous catalysts through the use of insoluble inorganic or organic carrier materials, such as silica, metal–organic frameworks, carbon nanomaterials and polymers [5,6,7]. The combination of homogeneous and heterogeneous catalysis offers distinct advantages, including the transfer of mechanistic knowledge derived from the defined molecular structure of the catalyst along with the benefits associated with the easier separation of products, reusability of the catalyst and the feasibility of integration into a flow reactor setup [5,8]. Moreover, this catalytic approach facilitates the circumvention of metal centers, which are already widely used as immobilized catalysts [9,10,11]. It has been shown that these metals exhibit less favorable environmental, toxicity, economic and sustainability properties [12]. Organocatalysis, which has evolved significantly in the recent decade, presents an alternative approach. Examples are known in which the functionalization of carrier materials such as silica or polyacrylonitrile by primary or tertiary amines is well suited for the catalysis of Knoevenagel, Mannich or Michael reactions and others under mild reaction conditions [8,13,14,15,16]. In addition to the nature of the catalyst, it has been demonstrated that the structure of the material is also of significant importance for efficient catalysis as well as a distinct porosity and a large surface area. The external morphological shape exerts a substantial impact on the accessibility of the catalytically active sites and therefore on the performance of the system [17,18]. In addition, to spheres, plates, sheets and cubes, fibers in particular offer the possibility of forming a three-dimensional structure without aggregation [19]. For instance, Li et al. demonstrated that catalysis of Knoevenagel reactions with amine-functionalized polyacrylonitrile threads resulted in considerable yields. However, these processes demanded a relatively substantial quantity of catalyst, as the configuration of the fibers did not optimally result in a large surface-to-volume ratio [13]. The suitability of a synthesis depends on its ability to produce a well-defined nanofibrous structure. In general, bottom-up bulk syntheses with or without template molecules and top-down methodologies have been shown to be appropriate.

Electrospinning, as an example of the latter, in particular, has garnered significant interest [19,20,21,22,23]. The fundamental setup of electrospinning consists of a high-voltage power supply, a metal needle as a spinneret, and a grounded conductor as a collector. The application of a voltage ranging from 1 to 30 kV results in the material solution undergoing electrostatic repulsion between the surface charges that are formed and a Coulomb force, which is a consequence of the electric field. These forces lead to the formation of a Taylor cone and upon exceeding forces stronger than the surface tension a long filamentous structure is extruded [24]. Electrospinning is therefore a simple, efficient, cost-effective and easily controllable method for synthesizing nanofibers by adjusting process parameters such as applied voltage, flow rate, needle-to-collector distance, material concentration and solution viscosity. It enables the synthesis of a wide variety of nanofiber materials composed of metal oxides, carbon-based materials, polymers and composites [25]. Combining this technique with polymeric materials confers several distinct advantages. The immobilization of catalytically active molecules, such as tertiary amines, can be achieved directly by polymerization. Subsequent processing of the polymeric solution results in the formation of a fibrous structure [26,27]. Furthermore, post-crosslinking can be employed to achieve hierarchical porosity, thereby resulting in a significantly enlarged surface area. In addition to the formation of large pores by the overlapping nanofibers, a wide-meshed polymer network can be achieved in the swollen state, resulting in optimal accessibility of the catalytically active centers [27,28].

Malononitrile derivatives represent a class of significant importance within the fields of organic synthesis and retrosynthesis, finding application in a diverse array of disciplines including pharmaceutical research, biotechnology, perfumery and electrochemical applications [29,30,31,32]. It is therefore pivotal to manufacture these derivatives in the most environmentally friendly manner possible, in accordance with the principles of green chemistry previously mentioned [33]. The presence of an active methylene group in the structure of malononitrile suggests the possibility of performing condensation reactions with other compounds, such as aldehydes. It is well established that these Knoevenagel reactions are known to proceed reliably in the presence of a strong base [34].

**Figure 1:**
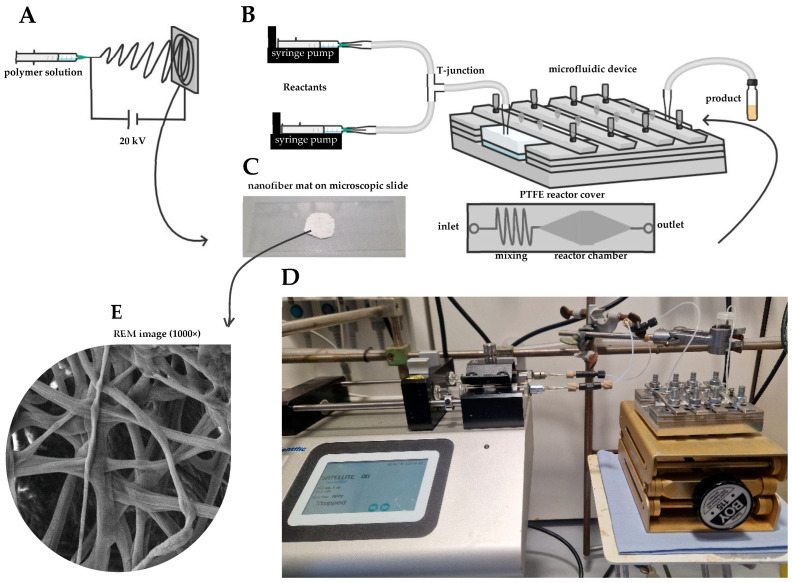
Overview of experimental setup. (**A**) Experimental electrospinning setup. (**B**) Schematic assembly of the microfluidic system. (**C**) Cut nanofiber mat on a microscopic glass slide and PTFE cover of the reactor with mixing loops and reactor chamber. (**D**) Experimental MFR setup. (**E**) SEM image with 1000× magnification of the nanofibrous structure.

For these reasons, the present work aims to synthesize aromatic and aliphatic malononitrile derivatives using a microfluidic reactor setup (MFR) and heterogeneous catalysis. In accordance with this objective, the initial step is the development and synthesis of polymer structures that are suitable for the immobilization of tertiary amines. Subsequently, these structures must undergo processing into nanofibers through electrospinning technique (Figure 1). The catalytically active polymer (CAP) is then subject to analysis via nuclear magnetic resonance (NMR) and Fourier-transformation infrared (FTIR) spectroscopy, complemented by scanning electron microscopy (SEM), energy-dispersive X-ray (EDX) (Supplementary Figures S36–S41) and elemental analyses (CHNS). This analytical approach enables the formulation of systematic investigations regarding the efficiency and turnover frequencies (TOF) of the developed heterogeneous catalyst system for the designated Knoevenagel reactions.

## 2. Results and Discussion

**Scheme 1:**
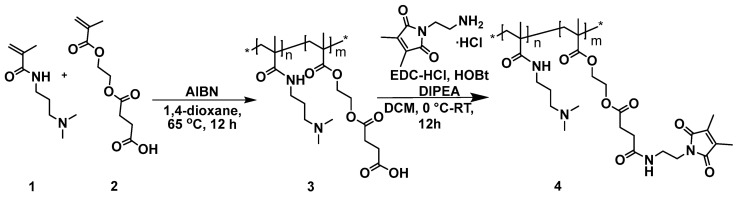
Synthesis of CAP Poly(DMAPMA-co-MME-DMMI) (**4**).

The catalytically active amine was immobilized by embedding it as a monomer within a polymeric structure. For this purpose, *N*-(3-(dimethylacrylamino)propyl)methacrylamide (DMAPMA, **1**) was polymerized via free radical polymerization initiated by AIBN, using mono-2-(methacryloyloxy)ethyl succinate (MMES, **2**) as a comonomer (Scheme 1). The resulting copolymer Poly(*N*-(3-(dimethylamino)propyl)methyacrylamide)-co-(mono-2-(methacryloyloxy)ethyl succinate) (Poly(DMAPMA-co-MMES), **3**) was subsequently modified through a carbodiimide-mediated amide coupling with the HCl salt of the photo crosslinker 1-(2-aminoethyl)-3,4-dimethyl-1*H*-pyrrole-2,5-dione. In this reaction, 1-ethyl-3-(3-dimethylaminopropyl)carbodiimide (EDC) was used to activate the carboxylic acid groups in the polymer side chain. The final CAP Poly(*N*-(3-(dimethylamino)propyl)methacrylamide)-co-(2-(methyacryloyloxy)ethyl 4-((2-(3,4-dimethyl-2,5-dioxo-2,5-dihydro-1*H*-pyrrole-1-yl)ethyl)amino)-4-oxobutanoate) (Poly(DMAPMA-co-MME-DMMI), **4**) was isolated, dried under high vacuum and stored under light-protected conditions. The characterization of polymers **3** and **4** was performed using ^1^H NMR spectroscopy and SEC. For Poly(DMAPMA-co-MMES) (**3**), NMR revealed a comonomer ratio of 5:1, while SEC analysis determined a molar mass of ${\bar{M}}_{n}$ = 59,000 g/mol. Poly(DMAPMA-co-MME-DMMI) (**4**) showed a molar mass of ${\bar{M}}_{n}$ = 98,000 g/mol. In both cases, the chemical structures were confirmed by comparison of their ^1^H NMR signals with those of the corresponding monomers (Supplementary Figures S1–S6).

**Figure 2:**
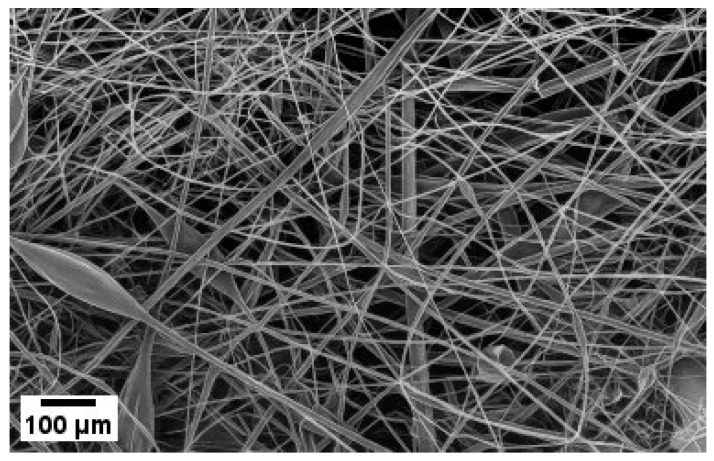
SEM images of nanofibers of **4** (1000×).

The synthesized polymer was processed into nanofiber mats using the electrospinning technique; however, the polymer alone was insufficient to form robust nanofiber mats. To overcome this limitation, a blend solution with a hydrophobic polymer was produced, that has been demonstrated to form continuous nanofibers during the electrospinning process. Polycaprolactone (PCL), a hydrophobic polymer known for generating stable fibers, was selected to stabilize the CAPs at a 1:1 weight ratio [35]. The polymer solution with a polymer weight percentage of 20% in THF/methanol (2:1) was loaded into a syringe with a stainless steel cannula. A syringe pump was utilized to maintain a constant flow rate of 0.5 mL/h, thereby ensuring a stable Taylor cone and adequate solvent evaporation. A voltage of 20 kV was applied in a horizontal arrangement with a needle-to-collector distance of 10 cm. This facilitated the successful fabrication of stable, uniformly thin nanofibers of polymer **4**, thereby circumventing significant beading and defects [36]. Figure 2 shows a SEM image of the manufactured structures at a magnification of 1000× immediately after the electrospinning process. The investigation revealed the presence of isolated nanofibers in the sample.

**Figure 3:**
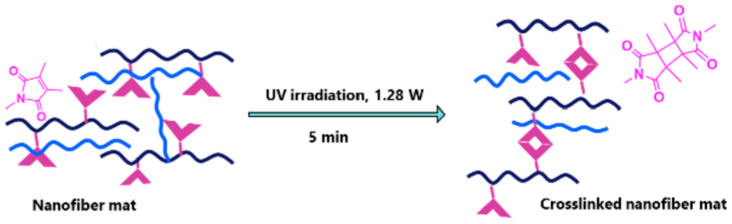
Schematic photo-crosslinking for formation of a polymeric nanofiber network.

Subsequent to the manufacturing process, the nanofiber mats were stored in conditions that prevented exposure to light. Immediately before utilization in catalysis within the microfluidic device, the mats were subjected to cross-linking through UV irradiation at a power of 1.28 W for a duration of 5 min. The photo-crosslinker, which contains 3,4-dimethylmaleimide functionalities, facilitates the formation of a cyclobutene rings between the polymeric side chains via a [2 + 2] cycloaddition, thereby creating a polymer network (Figure 3).

**Figure 4:**
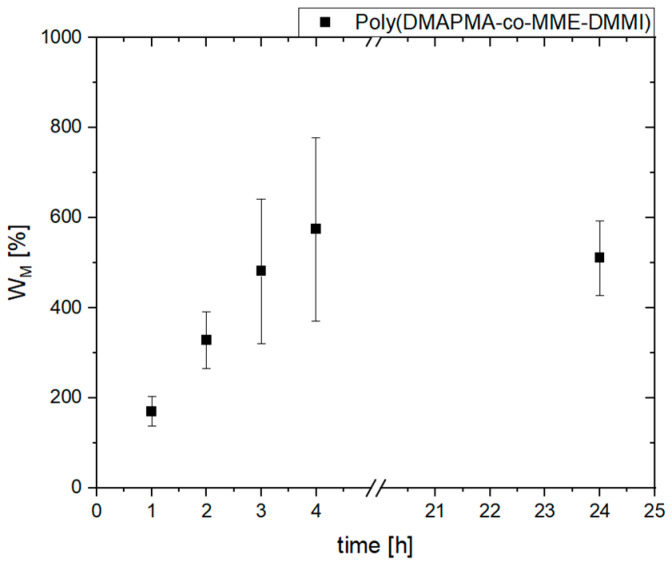
Swelling properties of nanofiber mat **4**.

The accessibility of the catalytically active centers has been demonstrated to be a primary factor in the efficiency of the nanofiber mats produced. It is evident that an increase in accessibility will result in a corresponding rise in the number of reactions that can be catalyzed in a given time period [37]. In addition to the surfaces of the nanofibers, further amino groups can be accessed via diffusion into the wide-meshed network of the polymer. The absorption of solvent and reactant molecules is influenced by the polymer’s property of swelling [38,39]. In order to investigate these swelling properties of the polymers in detail, swelling studies were carried out in the reaction solvent mixture DMSO:iPrOH (1:1) at room temperature. At designated time intervals of 1 h, 2 h, 3 h, 4 h and 24 h, the gels were weighted, and light microscopic images were recorded. Examining the results, after 24 h, gels of Poly(DMAPMA-co-MME-DMMI) (**4**) demonstrated an average percentage of solvent uptake (W_M_) of 510%. The observed swelling properties demonstrated that the photo-crosslinking process was successful, yielding hierarchical porous mats (Figure 4, Supplementary Table S1 and Figure S25).

**Figure 5:**
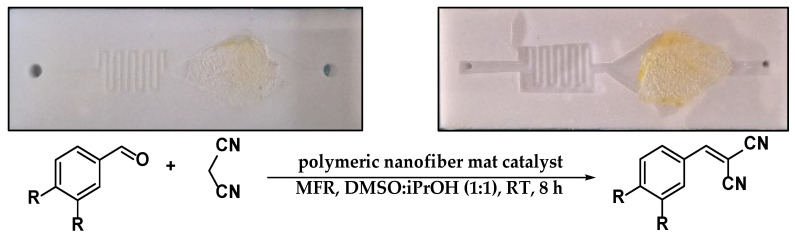
Synthesis of malononitrile derivatives by Knoevenagel reactions in presence of heterogeneous polymeric nanofiber mat **4** catalysts in MFR.

For comparison between the developed catalytic system and a previously developed system, which was also based on a tertiary amine as the catalytically active monomer and synthesized as dot structures on a micrometer scale using photolithography, the same reaction conditions were utilized in the subsequent catalysis experiments. The reactions were therefore carried out with the same diamond-shaped reactor chamber, in DMSO:iPrOH (1:1) as the solvent, at room temperature and with a reaction time of 8 h (Figure 5). As both the catalytically active monomer in this study and the reference system are tertiary amines, it can be deduced that a pK_a_ value of approximately 9 can be expected for both, resulting in a comparable base strength [40,41].

In order to use an equivalent quantity of catalyst in the reactions in comparison to the previous studies of catalytically active gel dots, elemental analysis was conducted on polymers of mat **4** [40]. The elemental composition was determined twice with particular focus placed on the nitrogen content. The proportion of catalytically active monomer per mass of the nanofiber mat could then be calculated using the average nitrogen content of 11.3% and the copolymer ratio of 5:1, determined by NMR analysis. This resulted in a comparable catalyst content of 3.6 μmol for a mat measuring 1.2 × 1.4 × 0.006 cm in the reactor.

The microfluidic system was essentially constructed with a syringe pump, two syringes containing reactant solutions, a T-junction, a diamond-shaped PTFE reactor chamber including mixing loops, and a vial for collecting the product. Before starting the catalysis, the nanofiber mats were cut to size and placed on a microscopic glass slide. The reactor was closed and sealed with Parafilm, after which it was placed in the aluminum carrier. All components were connected with PTFE tubes (iD = 0.2 mm). For pre-swelling of the polymeric mats, the flow rate was adjusted to 4 μL/min. The polymer mats were pre-swollen in the DMSO:iPrOH (1:1) reaction solvent mixture at a flow rate of 4 μL for a duration of at least 2 h, with the objective of optimizing the accessibility of the catalytically active centers from the initial stage. Subsequently, the reaction solutions with a concentration of 2 M containing malononitrile or aldehyde, were loaded into the syringes. The MFR reactions were then carried out for a duration of 8 h at a flow rate of 2 μL/min. NMR yields were determined using offline ^1^H NMR spectroscopy based on the signals of the aldehyde proton at approximately 10 ppm and the vinyl proton in the respective products at approximately 8 ppm (Table 1.). Furthermore, the malononitrile derivates were isolated and analyzed using ^1^H and ^13^C NMR spectroscopy, with a focus on identifying them correctly in the reaction mixture (Supplementary Figures S7–S18).

As illustrated in Table 1, the nanofiber mat **4**, which is based on Poly(DMAPMA-co-MME-DMMI), has attained notably higher NMR yields in comparison to the established catalyst system (column 4), which also contains a tertiary amine as the catalytically active monomer. While electron-deficient aromatics achieved particularly high NMR yields in previous studies [35], the present work focused specifically on less reactive, electron-rich substrates and aliphatic aldehydes, which are generally less reactive. It is notable that especially reactions 1 and 4 demonstrated promising results, with a doubling of the NMR yield. These aromatic substrates with electron-donating substituents generally exhibit lower conversion rates in Knoevenagel reactions, however in this study, entries 2 and 4 demonstrated well turnovers of at least 50% [42]. It was found that slightly lower turnovers were achieved with the non-aromatic aldehyde as reactant, as would be expected.

In order to compare the results with other literature values, the turnover frequencies (TOF) for the reactions carried out with the nanofiber mats **4** were calculated. The TOF values, which range from 52 to 79 h^−1^, are found to be 3–5 times higher than in the gel dots comparison system (Table 2) [40]. In addition, Li et al. have used tertiary amine-functionalized polyacrylonitrile fibers to catalyze Knoevenagel reactions for the synthesis of malononitrile derivatives. These comparable systems achieved a TOF of 6 h^−1^ in the reaction of malononitrile with benzaldehyde, which indicates that both the heterogeneous catalyst and the microfluidic setup are highly efficient [13].

In addition to the catalytically active centers accessible by swelling, the active centers on the surface of the structures are crucial for the observed catalytic effects [43]. Therefore, the surface area of the nanofibers was calculated using SEM images. This was performed by determining the thickness at six positions and the known dimensions of the mats used. Analysis of the results indicates an average fiber thickness of 2.97 ± 0.79 μm, which results in a surface are 35-times larger than the reference system with 158 gel dots [40]. This increase in surface area is the reason for the significantly higher TOFs and thus the higher efficiency of the catalytic system.

**Figure 6:**
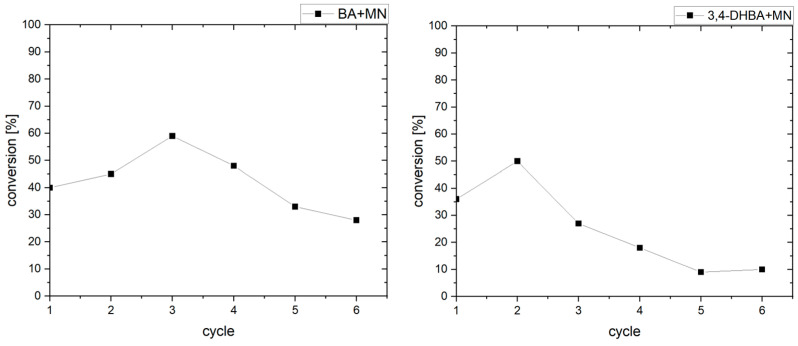
NMR yields of nanofiber mat **4** in the reaction of benzaldehyde with malononitrile in six reaction cycles (**left**) and in the reaction of 3,4-dihydroxybenzaldehyde with malononitrile in six reaction cycles (**right**).

**Figure 7:**
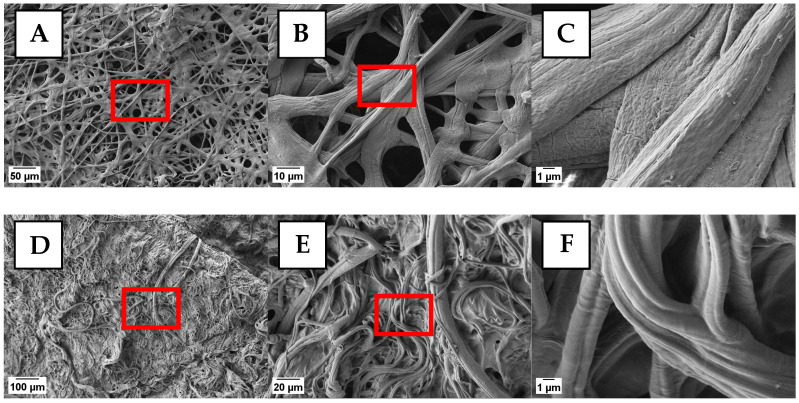
(**A**–**C**) Nanofiber mat **4** after crosslinking with magnifications of 200×, 1000× and 5000×; (**D**–**F**) Nanofiber mat **4** after 9 cycles of catalysis with magnifications of 100×, 500× and 5000×.

As a fundamental principle of green chemistry, the reusability of a catalyst system is also a crucial factor. In order to investigate the possibility of repeated use of a catalyst mat, the reactor was rinsed with DMSO:iPrOH (1:1) at a flow rate of 4 μL/min for 2 h post-reaction. Following this, the mat was then used for a further reaction. The nanofiber mat **4** showed reusability but also a decrease in NMR yields after two or three cycles, respectively, yielding a NMR yield of 30% in the reaction of benzaldehyde in the sixth cycle, and a yield of 10% in the reaction of 3,4-dihydroxybenzaldehyde (Figure 6, Supplementary Figures S34 and S35). Despite the observed decline, the NMR yields remain comparable to, if not superior to, those of the comparison system of gel dots [40]. To investigate the cause of the decrease, FTIR spectra were taken before and after the sixth reaction, as previous studies have shown that this allows conclusions to be drawn about reusability [18]. No significant chemical differences were identified that could have caused this decrease in NMR yields (Supplementary Figures S19–S22). Consequently, additional SEM images of the nanofiber mats **4** were recorded following crosslinking and after 9 cycles of catalysis (Figure 7).

In comparison with Figure 2, which shows the nanofibers immediately following electrospinning, Figure 7A–C demonstrates a connection of overlapping fibers after the crosslinking process. Furthermore, there are minor regions that exhibit slight film formation. Figure 7D–F presents the nanofiber mat **4** after 9 cycles of catalysis. Significant transformations in the structure are visible.

The fibers are no longer separated and are rudimentary outlines on a continuous film. Film formation of PCL fibers has also been observed in other studies, due to the influence of solvents [44]. The result of this process is a significant decrease in accessible surface area and, consequently, in the number of catalytically active centers that can be reached. This phenomenon explains the observed decrease in NMR yields with increasing catalysis cycles, thus pointing to the need for identification of alternative polymers that can stabilize the nanofibers under the influence of solvents (Supplementary Figures S26–S31). Therefore, this study highlighted the enormous impact of the structural properties on the efficiency of heterogeneous catalysts in microfluidic systems.

## 3. Conclusions

The development of efficient catalysts is a fundamental principle of green chemistry, a field which is gaining increasing attention in the movement towards sustainability in chemical research. In this study, a catalytically active polymer based on a tertiary amine (**4**) was developed and structured by electrospinning in the form of nanofibers with PCL as a reinforcing additive. Subsequent photo-crosslinking resulted in a polymer network with the ability to swell and hierarchical porosity. The nanofiber mats were then utilized in microflow reactions for the synthesis of malononitrile derivatives. The NMR yields of the reactions were determined using offline ^1^H NMR spectroscopy, and the isolated products were identified using ^1^H and ^13^C NMR spectroscopy, mass spectroscopy and melting point analysis. Nanofiber mats **4** exhibited elevated turnovers of 40–60% and TOFs of 50–80 h^−1^, which were consistently higher than those of comparable, already established systems [13,40]. Finally, the turnover development was observed in six catalysis cycles. FTIR and SEM analyses attributed the noticeable decrease in NMR yields to film formation of the PCL components of the nanofibers, which means that this is less suitable as an additive for long-term stabilization. Furthermore, SEM images revealed the substantial surface area of the produced nanofibers. This in combination with the swelling properties lead to a high accessibility of catalytically active sites and therefore the particular efficiency of the developed heterogeneous catalysts used in combination with the MFR process, which intrinsically already improves synthesis efficiency.

## 4. Materials and Methods

### 4.1. Materials

*N*-(3-Dimethylaminopropyl)-*N*’-ethylcarbodiimide hydrochloride (EDC-HCl) (98%) was purchased from ABCR GmbH (Karlsruhe, Germany). Hexafluoro-2-propanol (99%) was purchased from Carbolution (St. Ingbert, Germany). Azobis(isobutyronitrile) (AIBN) (98%) and 1-hydroxybenzotriazole (HOBt) (98%) were obtained from ThermoFisher Scientific (Waltham, MA, USA). Malononitrile (99%), 4-methoxy benzaldehyde (98%), mono-2-(methacryloyloxy)ethyl succinate with 750 ppm monomethyl ether hydroquinone, *N*,*N*-diisopropylethylamin (99%), *N*-[3-(dimethylamino)propyl]methacrylamide (99%) with MEHQ, polycaprolactone (PCL) (M_n_ = 45,000 g/mol) and potassium trifluoroacetate (98%) were purchased from Sigma Aldrich (Taufkirchen, Germany). Dimethylsulfoxide (DMSO) (99.9%) and 1,4-dioxane (99.5%) were purchased from Grüssing GmbH (Filsum, Germany). Benzaldehyde (98%), 3,4-dihydroxy benzaldehyde (98%) and 4N hydrogenchloride in 1,4-dioxane were purchased from Tokyo Chemical Industries (TCI, Eschborn, Germany). Deuterated chloroform (CDCl_3_) (99.8%) + Ag, deuterated dimethyl sulfoxide (DMSO-D_6_) (99.8%) and deuterium oxide (D_2_O) (99.9%) were obtained from Deutero GmbH (Kastellaun, Germany). Isobutyraldehyde was purchased from Merck (Darmstadt, Germany). Aqueous ammonia (25%), dichloromethane (DCM) (technical grade), diethyl ether (technical grade), *n*-hexane (technical grade), methanol (technical grade), 2-propanol (iPrOH) (technical grade), tetrahydrofuran (THF) (technical grade) and sodium chloride were obtained from Stockmeier Chemie (Bielefeld, Germany). The photo-crosslinker 1-(2-aminoethyl)-3,4-dimethyl-1*H*-pyrrole-2,5-dione was synthesized according to a reported procedure [45].

### 4.2. Characterizations and Instruments

^1^H Nuclear Magnetic Resonance (NMR) spectra were recorded using the Avance500 of Bruker (Billerica, MA, USA) at 500 MHz or at the Ascent700 of Bruker (Billerica, MA, USA) at 700 MHz. ^13^C NMR spectra were recorded at the Ascent700 at 176 MHz. 5 mg of the samples were dissolved in 500–600 μL of deuterated solvents (CDCl_3_, D_2_O or DMSO-d_6_) in NMR tubes. The prepared samples were scanned in the NMR spectrometer and the obtained data were analyzed by the TopSpin 4.5.0 software of Bruker (Billerica, MA, USA). The NMR signals were recorded with respect to the reference signals of the respective solvent. The electrospray ionization mass spectrometry (ESI-MS) was performed on the Synapt-2G HMDS of Waters (Milford, MA, USA) with a quadrupole time-of-flight (TOF) analyser. The samples were dissolved in acetonitrile with a concentration of 1 mg/mL. The melting point determination was conducted with the BÜCHI Melting Point B-545 with a heating rate of 1 °C/min. Size-exclusion chromatography (SEC) was carried out for molar weight determination in hexafluoro-2-propanol containing potassium trifluoroacetate (0.05 M) and BHT used as the internal standard with a flow rate of 1 mL/min. The setup consists of a precolumn PSS-PFG guard 7 μm, subsequent columns PSS-PFG 10^2^ Å 7 μm and PSS-PFG 10^3^ Å 7 μm, a Shodex RI-101 RI detector (Resonac Europe GmbH, Wiesbaden, Germany) and a LC-6200 pump of Merck (Darmstadt, Germany). The samples were prepared with a concentration of 6 mg/mL. The calibration was made with a PMMA standard. Attenuated total reflexion (ATR) infrared spectra were recorded on the VERTEX 70 FTIR instrument of Bruker (Billerica, MA, USA) at room temperature. All spectra were measured with a resolution of 4 cm^−1^ in the 4500–350 cm^−1^ region. Electrospinning was conducted in a custom-made setup consisting of a 30 kV high-voltage power supply unit (Scientific Instruments GmbH, Gilching, Germany), a syringe pump 11 Elite (Harvard Apparatus, Holliston, MA, USA) placed in a shielded and ventilated chamber. An Al foil covered plate was used as target. Photopolymerization was carried out using the Omnicure^®^ S1500 UV lamp from Lumen Dynamics (Mississauga, ON, Canada). For microscopic images of the gels a light microscope of Hund (Wetzlar, Germany) was used with a cold light source FLQ 150 M. The setup was combined with the camera iDS uEye UI146xLE-C and the iDS uEye cockpit 4.91.0 software (iDS, Obersulm, BW, Germany). For determination of the dimension of the gels a microscopic ruler of PYSER-SGI Ltd. (Edenbridge, UK) was used. Scanning electron microscopy (SEM) images were recorded with the Zeiss Neon 40 (Oberkochen, Germany) with a SE2 detector and a Schottky field emitter. The acceleration voltage was kept between 2.00 and 10.00 kV and the sample current between 4 pA and 20 nA to obtain a magnification of 100–5000×. Energy-dispersive X-ray spectroscopy was performed with the Zeiss Neon 40 (Oberkochen, Germany) with an ultra-dry detector of Thermo Fisher Scientific (Waltham, MA, USA). In the point & shoot modus an acceleration voltage of 10.0 kV was used. For sample preparation, the gel mats were attached to a carbon pad and sputtered with 5 nm gold-palladium. The elemental analysis (CHNS) was performed using a vario MicroCube analyser of Elementar (Langenselbold, Germany). The microfluidic reactor was assembled according to the previous reported procedure [46]. The microfluidic reactor system consists of two 5 mL Hamilton 1000 series syringes (Hamilton, Bonaduz, Switzerland), a Legato 200 syringe pump (KD Scientific, Holliston, MA, USA), a reactor and a collecting product vial. The reactor has a diamond-shaped reaction chamber and mixing loops imprinted on a PTFE layer covered by a microscopic glass slide containing the polymeric nanofiber mats (1.2 × 1.4 × 0.006 cm). The microfluidic reactor was sealed with a parafilm layer and fixed in a self-made aluminum holder with screws at a torque of 0.8 Nm. The syringes were connected by PTFE capillary tubes and a T-junction (iD = 0.2 mm) (Fisher Scientific, Waltham, MA, USA) and fed into the reactor.

### 4.3. Synthesis of CAP

(a) Synthesis of Poly(DMAPMA-co-MME-DMMI) (**4**)

*N*-[3-(dimethylamino)propyl] methacrylamide (21.7 mmol) and mono-2-(methacryloy loxy)ethyl succinate (2.94 mmol) were taken in a 25 mL pear-shaped flask and shield with a septum. After dissolving the monomers in 10 mL of 1,4-dioxane, the reaction mixture was purged with nitrogen gas for 15 min. AIBN (0.3 mmol) was dissolved in 3 mL 1,4-dioxane and added to the reaction mixture, which was purged afterwards with nitrogen gas again for 5 min. The reaction mixture was stirred at 65 °C for 12 h, after which the polymer was precipitated in 100 mL of *n*-hexane for three times and dried under high vacuum to obtain **3** (2.30 g).

^1^H NMR (500 MHz, DMSO-d6): δ (ppm) = 0.85–0.91 (m, 6H), 1.16 (m, 2H), 1.28 (m, 2H), 1.58 (m, 2H), 1.75 (m, 2H), 1.89 (m, 2H), 2.15 (s, 6H), 2.40 (s, 2H), 3.08 (s, 2H), 3.81 (m, 2H), 4.23 (m, 2H), 7.59 (s, 1H). SEC: ${\bar{M}}_{n}$ = 59,000 g/mol, ${\bar{M}}_{w}$ = 170,000 g/mol, $\bar{D}$ = 2.99.

Furthermore, **3** (2.30 g) was taken in a 100 mL round-bottom flask, dissolved in 40 mL of DCM, and cooled to 0 °C using an ice bath. To the reaction mixture, 1-(2-aminoethyl)-3,4-dimethyl-1*H*-pyrrole-2,5-dione (2.93 mmol), EDC-HCl (3.00 mmol), HOBt (3.00 mmol) and DIPEA (3.50 mmol) were added and stirred at room temperature for 12 h in the dark. After completion of the reaction the product was precipitated in *n*-hexane five times and dried under vacuum to obtain **4** (2.04 g) as a sticky colorless solid. The polymer was stored in a dark place.

^1^H NMR (500 MHz, D_2_O): δ (ppm) = 0.91 (m, 3H), 1.31 (m, 2H), 1.65 (s, 2H), 1.81 (m, 3H), 1.98 (s, 2H), 2.09 (s, 6H), 2.47 (s, 6H), 2.63 (m, 2H), 2.78 (m, 2H), 3.24 (s, 2H), 3.29 (s, 6H), 3.55 (m, 2H), 3.75 (m, 2H), 3.87 (m, 2H), 4.41 (m, 2H), 5.24 (s, 1H). SEC: ${\bar{M}}_{n}$ = 98,000 g/mol, ${\bar{M}}_{w}$ = 370,000 g/mol, $\bar{D}$ = 3.77.

### 4.4. Synthesis of Nanofiber Mats by Electrospinning

The synthesized polymer **4** was used to prepare nanofiber mats using the electrospinning technique. PCL was added at a ratio of 1:1 to strengthen the fibers and form peelable polymer mats that can be transferred to a flow reactor after electrospinning. Initially, polymer **4** was dissolved in methanol (30 wt.%) and PCL in THF (30 wt.%) and both solutions were stirred overnight to produce homogeneous solutions. The blend solution was then prepared by mixing 0.5 mL of the catalytic polymer solution and 0.5 mL of the PCL solution, followed by dilution with 0.5 mL of THF and stirring for 2 h. The blend solution was drawn into a syringe containing a stainless-steel cannula. This was fixed to a syringe pump to maintain a constant flow rate of 0.5 mL/h, and was connected to the generator. The fibers were generated using electrospinning in a horizontal arrangement with a needle-to-generator distance of 10 cm in the presence of an applied voltage of 20 kV.

Nanofiber mat **4** as synthesized:

FTIR: ṽ (cm^−1^) = 3281 v(N-H), 3095 v(=C-H), 2945–2831 v(C-H), 1724 v(C=O), 1645 v(C=O), 1556 δ(N-H), 1470–1365 δ(C-H), 1294 v(C-N), 1238–1167 v(O=C-O-C), 1045 δ(=C-C-H).

The photocrosslinker 1-(2-aminoethyl)-3,4-dimethyl-1*H*-pyrrole-2,5-dione was incorporated into the polymer structure in order to crosslink the polymeric nanofibers following electrospinning. The polymer mats were therefore irradiated for 5 min at an intensity of 1.28 W immediately before they were used as a heterogeneous catalyst, using the Omnicure^®^ S1500 UV lamp), with a distance of 8 cm between the sample and the lamp.

Nanofiber mat **4** after crosslinking:

FTIR: ṽ (cm^−1^) = 3265 v(N-H), 2949–2785 v(C-H), 1724 v(C=O), 1641 v(C=O), 1554 δ(N-H), 1468–1385 δ(C-H), 1290 v(C-N), 1263–1175 v(O=C-O-C).

### 4.5. Swelling Studies of the Nanofiber Mats

The percentage of solvent uptake W_M_ was determined by weighing the polymer mats before and after leaving them in the reaction solvent mixture DMSO:iPrOH (1:1) for a set period of time. Small pieces of the nanofiber mats were cut and weighed. After residence times of 1 h, 2 h, 3 h, 4h, as well as 24 h, in the swelling chamber at a defined temperature of 20 °C, which was controlled by a Julabo F12+ED thermostat (Julabo, Seelbach, Germany), the weight of the samples was determined and light microscopic images were taken.

(1) $$W_{M}=\frac{w_{2}-w_{1}}{w_{1}}\cdot 100\%$$

$$W_{M}=\mathrm{percentage}\mathrm{of}\mathrm{solvent}\mathrm{uptake}\;[\%]$$

$$w_{1}=\mathrm{weight}\mathrm{of}\mathrm{the}\mathrm{dry}\mathrm{gel}\mathrm{mat}\;[g]$$

$$w_{2}=\mathrm{weight}\mathrm{of}\mathrm{the}\mathrm{swollen}\mathrm{gel}\mathrm{mat}\;[g]$$

Triple determinations were carried out.

Nanofiber mat **4** after swelling:

FTIR: ṽ (cm^−1^) = 3286 v(N-H), 2943–2866 v(C-H), 1722 v(C=O), 1642 v(C=O), 1564 δ(N-H), 1472–1365 δ(C-H), 1294 v(C-N), 1238–1164 v(O=C-O-C).

### 4.6. Assembly of Microfluidic Reactor and Microfluidic Reactions

Following assembly, the polymeric mats were pre-swollen for at least 2 h in the reaction solvent mixture DMSO:iPrOH (1:1) at a flow rate of 4.0 μL/min. The syringes were then loaded with the respective reactant stock solutions, each with a concentration of 2 M (1.92 mmol, 960 μL) of either malononitrile or aldehyde. The flow rate was adjusted to 2 μL/min for an 8 h reaction time. NMR yield was determined by offline ^1^H NMR spectroscopy by comparison of the product peak singlet around 8 ppm and the aldehyde signal around 10 ppm. Therefore, the integral of the product peak was divided by the sum of the integral of the aldehyde peak and the integral of the product peak. All the MFR reactions were performed similarly. The spectral data are given in the Supplementary Data (Figures S7–S18, S34 and S35).

To isolate the products, the reaction mixtures were diluted with 10 mL of diethyl ether and washed five times with 10 mL of water. The organic phase was then separated and concentrated by rotary evaporation. Finally, the isolated products were dried under high vacuum to obtain the pure products.

(a) Synthesis of 2-benzylidene malononitrile

The product was isolated as a white powder. MP: 81.5–82.9 °C. ^1^H NMR (700 MHz, DMSO-d6): δ (ppm) = 7.63 (t, 2H, ^3^J_HH_ = 7.77 Hz), 7.70 (t, 1H, ^3^J_HH_ = 7.42 Hz), 7.96 (d, 2H, ^3^J_HH_ = 7.35 Hz), 8.56 (s, 1H). ^13^C NMR (176 MHz, DMSO-d6): δ (ppm) = 81.6, 113.2, 114.2, 129.5, 130.5, 131.3, 134.4, 161.6. ESI-MS (m/z): C_10_H_6_N_2_ [M-H] ^+^. Mass calculated: 154.0531 Da. Mass found: 153.9857 Da.

(b) Synthesis of 2-(4-methoxybenzylidene) malononitrile

The product was isolated as a yellow crystalline solid. MP: 113.4–115 °C. ^1^H NMR (700 MHz, DMSO-d6): δ (ppm) = 3.89 (s, 3H), 7.19 (d, 2H, ^3^J_HH_ = 8.94 Hz), 7.98 (d, 2H, ^3^J_HH_ = 8.89 Hz), 8.40 (s, 1H). ^13^C NMR (176 MHz, DMSO-d6): δ (ppm) = 55.9, 76.9, 113.9, 114.8, 115.2, 124.1, 133.4, 160.5, 164.4. ESI-MS (m/z): C_11_H_8_N_2_O [M]^+^. Mass calculated: 184.0637 Da. Mass found: 184.0632 Da.

(c) Synthesis of 2-(2-methylpropylidene) malononitrile

The product was isolated as a yellow liquid. ^1^H NMR (700 MHz, DMSO-d6): δ (ppm) = 1.11 (d, 6H, ^3^J_HH_ = 6.65 Hz), 2.84 (m, 1H), 7.81 (d, 1H, ^3^J_HH_ = 10.3 Hz). ^13^C NMR (176 MHz, DMSO-d6): δ (ppm) = 20.4, 32.7, 85.9, 111.2, 112.7, 177.3. ESI-MS (m/z): C_7_H_8_N_2_ [M-H]^+^. Mass calculated: 120.0687 Da. Mass found: 120.0589 Da.

(d) Synthesis of 2-(3,4-dihydroxybenzylidene) malononitrile

The product was isolated as a yellow powder. MP 218.3–219.7 °C (Decomposition). ^1^H NMR (700 MHz, DMSO-d6): δ (ppm) = 6.92 (d, 1H, ^3^J_HH_ = 8.40 Hz), 7.33 (dd, 1H, ^3^J_HH_ = 8.40 Hz, ^4^J_HH_ = 2.24 Hz), 7.54 (d, 1H, ^4^J_HH_ = 2.24 Hz), 8.20 (s, 1H). ^13^C NMR (176 MHz, DMSO-d6): δ (ppm) = 20.4, 32.7, 85.9, 111.2, 112.7, 177.3. ESI-MS (m/z): C_10_H_6_N_2_O_2_ [M]^+^. Mass calculated: 185.0351 Da. Mass found: 185.0355 Da.

### 4.7. Reusability of Nanofiber Mats

After the reaction of malononitrile with the respective aldehyde, the microfluidic system was washed with the reaction solvent mixture DMSO:iPrOH (1:1) at a flow rate of 4 μL/min for 2 h. The microscopic glass slides containing the nanofiber mats were then removed for structural analysis of the gels using ATR-FTIR spectroscopy, as this method has previously been shown to determine the reusability of the gels [40]. The FTIR-spectra are provided in the Supplementary Data (Figures S19–S22).

Nanofiber mat **4** after reaction:

FTIR: ṽ (cm^−1^) = 3300 v(N-H), 2943–2868 v(C-H), 2204 v(CN), 1722 v(C=O), 1645 v(C=O), 1556 δ(N-H), 1472–1365 δ(C-H), 1294 v(C-N), 1238–1165 v(O=C-O-C).

### 4.8. Elemental Analysis (CHNS)

The vacuum-dried samples were weighted into a tin capsule and injected into a combustion tube in a fully automated process. After combustion at a temperature of 1150 °C, the combustion gases were separated in a helium gas stream in a temperature-programmed column and quantitatively determined at a thermal conductivity detector. The sample was measured with double determination.

Nanofiber mat **4:**

11.31 ± 0.28%N, 50.54 ± 0.06%C, 8.76 ± 0.09%H

### 4.9. Calculation of Turn over Frequency (TOF)

The following formular can be used to calculate the turn over frequency (TOF) of the catalyst [47].

(2) $$TOF=\frac{n(product)}{n(cat)\cdot t}$$

$$TOF=\mathrm{turn}\mathrm{over}\mathrm{frequency}\;[h^{-1}]$$

$$n\left(product\right)=\mathrm{amount}\mathrm{of}\mathrm{synthesized}\mathrm{product}\;[\mathrm{mol}]$$

$$n\left(cat\right)=\mathrm{amount}\mathrm{of}\mathrm{used}\mathrm{catalyst}\;[\mathrm{mol}]$$

t=reactiontime [h]

For determination of the amount of used catalyst, the average nitrogen weight percentage in the nanofiber mat **4** was measured using elemental analysis to 11.3%. Furthermore, the ^1^H NMR spectroscopy determined the ratio of the catalytic monomer to the crosslinker, both, both of which contain two nitrogen atoms, to be 5:1. Therefore, the catalyst content was calculated to be 28.2% of the weight of the nanofiber mat, based on the molecular mass of the catalytically active monomer and the weight percentage of the catalytically active nitrogen atom. This indicates that 3.6 μmol of catalyst was used for 1.92 mL of reactant solution.

## Figures and Tables

**Table 1:** NMR yields of microfluidic reactions for synthesis of malononitrile derivatives.

Reaction	Product	NMR Yields Nanofiber Mat Catalyst 4 [%] *	NMR Yields Gel Dots [%] [40] *
1	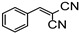	59	27
2	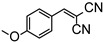	53	44
3	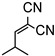	39	29
4	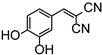	50	22

* Determined by ^1^H NMR.

**Table 2:** TOFs of microfluidic reactions for synthesis of malononitrile derivatives.

Reaction	Product	TOF [h^−1^] *
1	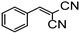	79
2	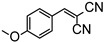	71
3	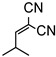	52
4	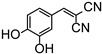	67

* Determined by elemental analysis (CHNS).

## Data Availability

The original contributions presented in this study are included in the article/Supplementary Materials. Further inquiries can be directed to the corresponding author.

## Supplementary Materials

The following supporting information can be downloaded at https://www.mdpi.com/article/10.3390/gels12010055/s1. Figure S1: Molecular structure of Poly(DMAPMA-co-MMES) (**3**); Figure S2: ^1^H NMR spectrum of Poly(DMAPMA-co-MMES) (**3**); Figure S3: SEC graph of Poly(DMAPMA-co-MMES) (**3**); Figure S4: Molecular structure of Poly(DMAPMA-co-MME-DMMI) (**4**); Figure S5: ^1^H NMR spectrum of Poly(DMAPMA-co-MME-DMMI) (**4**); Figure S6: SEC graph of Poly(DMAPMA-co-MME-DMMI) (**4**); Figure S7: Molecular structure of 2-benzylidene malononitrile; Figure S8: ^1^H NMR spectrum of 2-benzylidene malononitrile; Figure S9: ^13^C NMR spectrum of 2-benzylidene malononitrile; Figure S10: Molecular structure of 2-(4-methoxybenzylidene) malononitrile; Figure S11: ^1^H NMR spectrum of 2-(4-methoxybenzylidene) malononitrile; Figure S12: ^13^C NMR spectrum of 2-(4-methoxybenzylidene) malononitrile; Figure S13: Molecular structure of 2-(2-methylpropylidene) malononitrile; Figure S14: ^1^H NMR spectrum of 2-(2-methylpropylidene) malononitrile; Figure S15: ^13^C NMR spectrum of 2-(2-methylpropylidene) malononitrile; Figure S16: Molecular structure of 2-(3,4-dihydroxybenzylidene) malononitrile; Figure S17: ^1^H NMR spectrum of 2-(3,4-dihydroxybenzylidene) malononitrile; Figure S18: ^13^C NMR spectrum of 2-(3,4-dihydroxybenzylidene) malononitrile; Figure S19: FTIR spectrum of Poly(DMAPMA-co-MMES) (**4**) as synthesized; Figure S20: FTIR spectrum of Poly(DMAPMA-co-MMES) (**4**) after crosslinking; Figure S21: FTIR spectrum of Poly(DMAPMA-co-MMES) (**4**) after reaction of 3,4-dihydroxy benzaldehyde with malononitrile; Figure S22: FTIR spectrum of Poly(DMAPMA-co-MMES) (**4**) after swelling in DMSO:iPrOH (1:1) for 2 h; Figure S23: Light microscopic images of dry Poly(DMAPMA-co-MMES) (**4**) nanofiber mats with magnifications from 10x to 45x; Figure S24: Light microscopic images of wet (DMSO:iPrOH 1:1) Poly(DMAPMA-co-MMES) (**4**) nanofiber mats with magnifications from 10× to 45×; Table S1: Percentages of solvent uptake in DMSO:iPrOH (1:1) of nanofiber mats after 1 h, 2 h, 3 h, 4h and 24 h; Figure S25: Light microscopic images of swollen Poly(DMAPMA-co-MMES) (**4**) nanofiber mats after 1 h, 2 h, 3 h, 4 h and 24 h in DMSO:iPrOH (1:1) (10x); Figures S26–S28: SEM images of Poly(DMAPMA-co-MMES) (**4**) after crosslinking with magnification of 200× (Figure S26), 1000× (Figure S27) or 5000× (Figure S28); Figures: S29–S31: SEM images of Poly(DMAPMA-co-MMES) (**4**) after 9 cycles of heterogeneous catalysis with magnification of 100× (Figure S29), 1000× (Figure S30) or 5000× (Figure S31); Figures S32 and S33: REM images of Poly(DMAPMA-co-MMES) (**4**) after crosslinking (Figure S41) and after 9 cycles of heterogeneous catalysis (Figure S42), which were used to determine the thickness of the fibers; Figure S34: ^1^H NMR spectra of the reaction mixture of benzaldehyde with malononitrile after 8 h of reaction time in six cycles with the same Poly(DMAPMA-co-MME-DMMI) (**4**) nanofiber mat; Figure S35: ^1^H NMR spectra of the reaction mixture of 3,4-dihydroxy benzaldehyde with malononitrile after 8 h of reaction time in six cycles with the same Poly(DMAPMA-co-MME-DMMI) (**4**) nanofiber mat; Figure S36: Sample segments 1–4 of EDX spectroscopy analysis (left, magnification 27×); Sample segments 5–8 of EDX spectroscopy analysis (right, magnification 50x); Figure S37: EDX spectrum of sample segment 1; Figure S38: EDX spectrum of sample segment 2; Figure S39: EDX spectrum of sample segment 3; Figure S40: EDX spectrum of sample segment 4; Figure S41: EDX spectrum of sample segment 5; Figure S42: EDX spectrum of sample segment 6; Figure S43: EDX spectrum of sample segment 7; Figure S44: EDX spectrum of sample segment 8; Table S2: Wt.% and At.% results of EDX spectroscopy analysis of Poly(DMAPMA-co-MMES) (**4**).

## References

[1] Shakhashiri B.Z., Bell J.A. (2014). Climate Change and our responsibilities as chemists. Arab. J. Chem..

[2] Mitchell S., Martín A.J., Guillén-Gosálbez G., Pérez-Ramírez J. (2024). The Future of Chemical Sciences is Sustainable. Angew. Chem. Int. Ed..

[3] Colella M., Carlucci C., Luisi R. (2018). Supported Catalysts for Continuous Flow Synthesis. Top. Curr. Chem..

[4] Brivio M., Verboom W., Reinhoudt D.N. (2006). Miniaturized continuous flow reaction vessels: Influence on chemical reactions. Lab Chip.

[5] Chen T., Peng Y., Qui M., Yi C., Xu Z. (2023). Heterogenization of homogeneous catalysts in polymer nanoparticles: From easier recovery and reuse to more efficient catalysis. Coord. Chem. Rev..

[6] Fan Q.-H., Li Y.-M., Chan A.S.C. (2002). Recoverable Catalysts for Asymmetric Organic Synthesis. Chem. Rev..

[7] Yu X., Herberg A., Kuckling D. (2020). Micellar Organocatalysis Using Smart Polymer Supports: Influence of ThermoresponsiveSelf-Assembly on Catalytic Activity. Polymers.

[8] Turke K., Meinusch R., Cop P., Prates da Costa E., Brand R.D., Henss A., Schreiner P.R., Smarsly B.M. (2021). Amine-Functionalized Nanoporous Silica Monoliths for Heterogeneous Catalysis of the Knoevenagel Condensation in Flow. ACS Omega.

[9] Pullabhotla V.S.R.R., Rahman A., Jonnalagadda S.B. (2009). Selective catalytic Knoevenagel condensation by Ni-SiO_2_ supported heterogeneous catalysts: An environmentally benign approach. Catal. Commun..

[10] Kantam M.L., Choundary B.M., Reddy C.V., Rao K.K., Figueras F. (1998). Aldol and Knoevenagel condensations catalysed by modified Mg-Al hydrotalcite: A solid base as catalyst useful in synthetic organic chemistry. Chem. Commun..

[11] Calvino-Casilda V., Martín-Aranda R.M., López-Peinado A.J., Sobczak I., Ziolek M. (2009). Catalytic properties of alkali metal-modified oxide supports for the Knoevenagel condensation: Kinetic aspects. Catal. Today.

[12] Shaikh I.R. (2014). Organocatalysis: Key Trends in Green Synthetic Chemistry, Challenges, Scope towards Heterogenization, and Importance from Research and Industrial Point of View. J. Catal..

[13] Li G., Xiao J., Zhang W. (2011). Knoevenagel condensation catalyzed by a tertiary-amine functionalized polyacrylonitrile fiber. Green Chem..

[14] Schmiegel C.J., Berg P., Obst F., Schoch R., Appelhans D., Kuckling D. (2021). Continuous Flow Synthesis of Azoxybenzenes by Reductive Dimerization of Nitrosobenzenes with Gel-Bound Catalysts. Eur. J. Org. Chem..

[15] Killi N., Bartenbach J., Kuckling D. (2023). Polymeric Networks Containing Amine Derivatives as Organocatalysts for Knoevenagel Reaction within Continuously Driven Microfluidic Reactors. Gels.

[16] Killi N., Rumpke K., Kuckling D. (2025). Synthesis of Curcumin Derivatives via Knoevenagel Reaction Within a Continuously Driven Microfluidic Reactor Using Polymeric Networks Containing Piperidine as a Catalyst. Gels.

[17] Song W., Zhang Y., Tran C.H., Choi H.K., Yu D.G., Kim I. (2023). Porous organic polymers with defined morphologies: Synthesis, assembly, and emerging applications. Prog. Polym. Sci..

[18] Schmiegel C.J., Baier R., Kuckling D. (2021). Direct Asymmetric Aldol Reaction in Continuous Flow Using Gel-Bound Organocatalysts. Eur. J. Org. Chem..

[19] Liu R., Hou L., Yue G., Li H., Zhang J., Liu J., Miao B., Whang N., Bai J., Cui Z. (2022). Progress of Fabrication and Applications of Electrospun Hierarchically Porous Nanofibers. Adv. Fiber. Mater..

[20] Yang C., Wang K., Lyu W., Liu H., Li J., Wang Y., Jiang R., Yuan J., Liao Y. (2024). Nanofibrous Porous Organic Polymers and Their Derivatives: From Synthesis to Applications. Adv. Sci..

[21] Shi S., Si Y., Han Y., Wu T., Iqbal M.I., Fei B., Li R.K.Y., Hu J., Qu J. (2021). Recent Progress in Protective Membranes Fabricated via Electrospinning: Advanced Materials, Biomimetic Structures, and Functional Applications. Adv. Mater..

[22] Li D., Xia Y. (2004). Electrospinning of Nanofibers: Reinventing the Wheel?. Adv. Mater..

[23] Malara A. (2024). Is electrospinning a suitable technique to develop heterogeneous catalysts?. Chem. Eng. Res. Des..

[24] Cho Y., Baek J.W., Sagong M., Ahn S., Nam J.S., Kim I.D. (2025). Electrospinning and Nanofiber Technology: Fundamentals, Innovations, and Applications. Adv. Mater..

[25] Abdulhussain R., Adebisi A., Conway B.R., Asare-Addo K. (2023). Electrospun nanofibers: Exploring process parameters, polymer selection, and recent applications in pharmaceuticals and drug delivery. J. Drug Deliv. Sci. Technol..

[26] Zhang J., Yu M., Tao S. (2024). Advanced electrospinning nanomaterials: From spinning fabrication techniques to electrochemical applications. Nano Res..

[27] Xue J., Wu T., Dai Y., Xia Y. (2019). Electrospinning and Electrospun Nanofibers: Methods, Materials, and Applications. Chem. Rev..

[28] Metze F.K., Sant S., Meng Z., Klok H.A., Kaur K. (2023). Swelling-Activated, Soft Mechanochemistry in Polymer Materials. Langmuir.

[29] Kotowicz S., Sek D., Kula S., Fabiańczyk A., Siwy M., Filapek M., Szłapa-Kula A., Małecki J.G., Jonsson-Niedziółka M., Niedziółka-Jonsson J. (2018). Malonitrile derivatives as push-pull molecules: Structure-properties relationships characterization. J. Lumin..

[30] Wand S., Zhao Z., Wang Y., Zheng H., Zhang Q., Zhang L., Zhang J., Lu H., Dong Y. (2024). Design, Synthesis, and Bioactivity Determination of Novel Malonitrile Derivatives Containing 1,2,3-Triazole. J. Agric. Food Chem..

[31] Öztürk Kesebir A., Daĝalan Z., Güller P., Nişanci B., Irfan Küfrevioĝlu Ö. (2024). In vitro inhibition potency of malononitrile derivatives on the activity of two pentose phosphate pathway enzymes: Accompanied by molecular docking evaluation. Z. Naturforsch. C.

[32] Jadhav A.L., Yadav G.D. (2019). Clean synthesis of benzylidenemalononitrile by Knoevenagel condensation of benzaldehyde and malononitrile: Effect of combustion fuel on activity and selectivity of Ti-hydrotalcite and Zn-hydrotalcite catalysts. J. Chem. Sci..

[33] Hassan E.A., Elmaghraby A.M. (2015). The Chemistry of Malononitrile and its derivatives. Int. J. Innov. Sci. Res..

[34] Modal J., Modak A., Bhaumik A. (2011). Highly efficient mesoporous base catalyzed Knoevenagel condensation of different aromatic aldehydes with malononitrile and subsequent noncatalytic Diels-Alder reactions. J. Mol. Catal. A Chem..

[35] Azari A., Golchin A., Maymand M.M., Mansouri F., Ardeshirylajimi A. (2021). Electrospun Polycaprolactone Nanofibers: Current Research and Applications in Biomedical Application. Adv. Pharm. Bull..

[36] Bonakdar M.A., Rodrigue D. (2024). Electrospinning: Processes, Structures, and Materials. Macromol.

[37] Zhang Y., Wang B., Elageed E.H.M., Qin L., Ni B., Liu X., Gao G. (2016). Swelling Poly(ionic liquid)s: Synthesis and Application as Quasi-Homogeneous Catalysts in the Reaction of Ethylene Carbonate with Aniline. ACS Macro Lett..

[38] Xu F., Sheardown H., Hoare T. (2016). Reactive electrospinning of degradable poly(oligoethylene glycol methacrylate)-based nanofibrous hydrogel networks. Chem. Commun..

[39] Tsuge M., Takahasi K., Kurimoto R., Fulati A., Uto K., Kikuchi A., Ebara M. (2019). Fabrication of Water Absorbing Nanofiber Meshes toward an Efficient Removal of Excess Water from Kidney Failure Patients. Fibers.

[40] Berg P., Obst F., Simon D., Richter A., Appelhans D., Kuckling D. (2020). Novel Application of Polymer Networks Carrying Tertiary Amines as Catalysts Inside Microflow Reactors Used for Knoevenagel Reactions. Eur. J. Org. Chem..

[41] Rossini E., Bochevarov A.D., Knapp E.W. (2018). Empirical Conversion of pKa Values between Different Solvents and Interpretation of the Parameters: Application to Water, Acetonitrile, Dimethyl Sulfoxide, and Methanol. ACS Omega.

[42] Machado T.F., Valente A.J.M., Silva Serra M.E., Murtinho D. (2023). Diazo-coupled porous organic polymers as efficient catalysts for metal-free Henry and Knoevenagel reactions. Microporous Mesoporous Mater..

[43] Morgenthaler M., Schweizer E., Hoffman-Röder A., Benini F., Martin R.E., Jaeschke G., Wagner B., Fischer H., Bendels S., Zimmerli D. (2007). Predicting and Tuning Physicochemical Properties in Lead Optimization: Amine Basicities. ChemMedChem.

[44] Li H., Zhu C., Xue J., Ke Q., Xia Y. (2017). Enhancing the Mechanical Properties of Electrospun Nanofiber Mats through Controllable Welding at the Cross Points. Macromol. Rapid Commun..

[45] Rodin M., Helle D., Kuckling D. (2024). Pillar[5]arene-based dually crosslinked supramolecular gel as a sensor for the detection of adiponitrile. Polym. Chem..

[46] Simon D., Obst F., Haefner S., Heroldt T., Peiter M., Simon F., Richter A., Voit B., Appelhans D. (2019). Hydrogel/enzyme dots as adaptable tool for non-compartmentalized multi-enzymatic reactions in microfluidic devices. React. Chem. Eng..

[47] Prete P., Fiorentino A., Rizzo L., Proto A., Cuccuniello R. (2023). Open the way to turnover frequency determination in (photo)Fenton processes for catalytic activities comparison. Catal. Today.

