# Supplementary material for: Nanofiber Mats as Amine-Functionalized Heterogeneous Catalysts in Continuous Microfluidic Reactor Systems
Source: Gels. 2026 Jan 6;12(1):55. doi: 10.3390/gels12010055 (PMC12840762; doi:10.3390/gels12010055)
Supplement: Supplementary file 1 [file gels-12-00055-s001.zip › gels-4023636-supplementary.pdf]

Supporting information

Nanofiber Mats as Amine-Functionalized Heterogeneous Catalysts in Continuous Microfluidic Reactor Systems

Katja Rumpke[1], Naresh Killi[1], Barbara Dittrich[2], Andreas Herrmann[2,3] and Dirk Kuckling[1]

[1] Department of Chemistry, Paderborn University, Warburger Str. 100, 33098 Paderborn, Germany; krumpke@mail.uni-paderborn.de (K.R.); naresh.killi@uni-paderborn.de (N.K.)

[2] DWI - Leibniz-Institut für Interaktive Materialien e.V., Forckenbeckstr. 50, 52074 Aachen, Germany; dittrich@dwi.rwth-aachen.de (B.D.)

[3] Institute for Technical and Macromolecular Chemistry, Rheinisch-Westfälische Technische Hochschule (RWTH) Aachen University, 52074 Aachen, Germany; herrmann@dwi.rwth-aachen.de (A.H.)

* Correspondence: dirk.kuckling@uni-paderborn.de (D.K.)

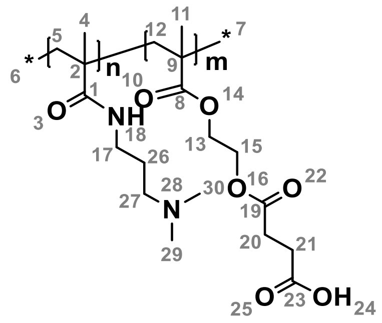

Figure S1: Molecular structure of Poly(DMAPMA-co-MMES) (**3**)

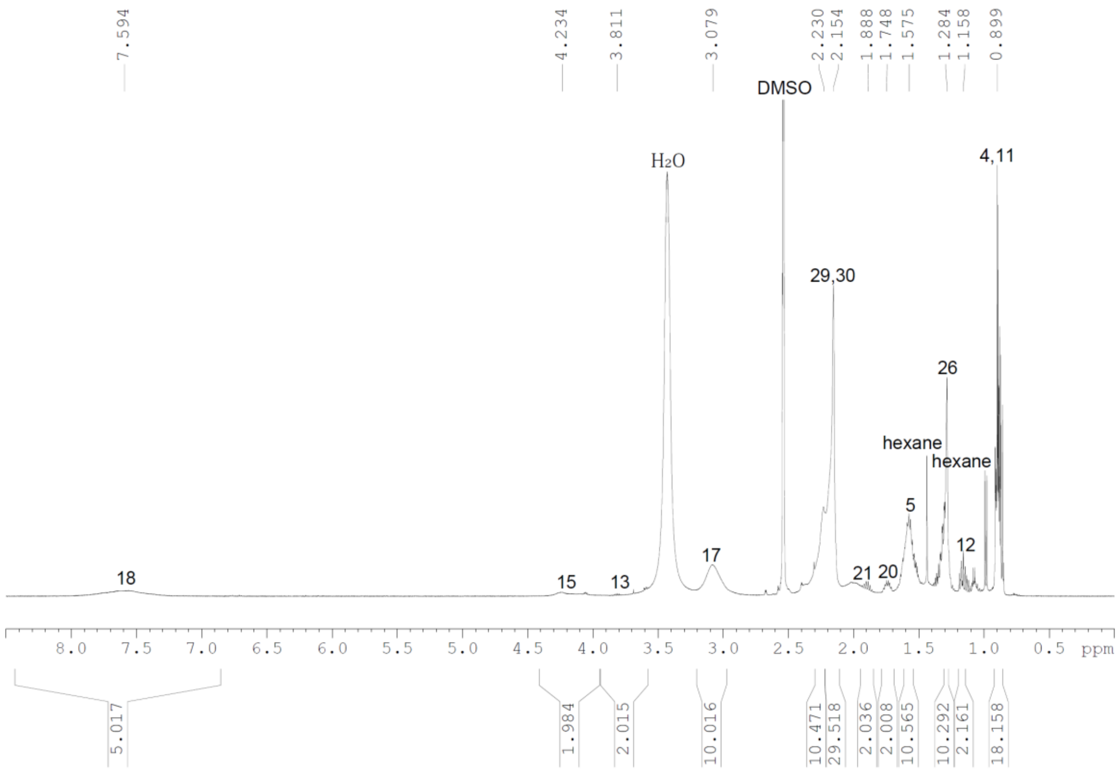

Figure S2: ^1H NMR spectrum of Poly(DMAPMA-co-MMES) (**3**) in DMSO-d_6

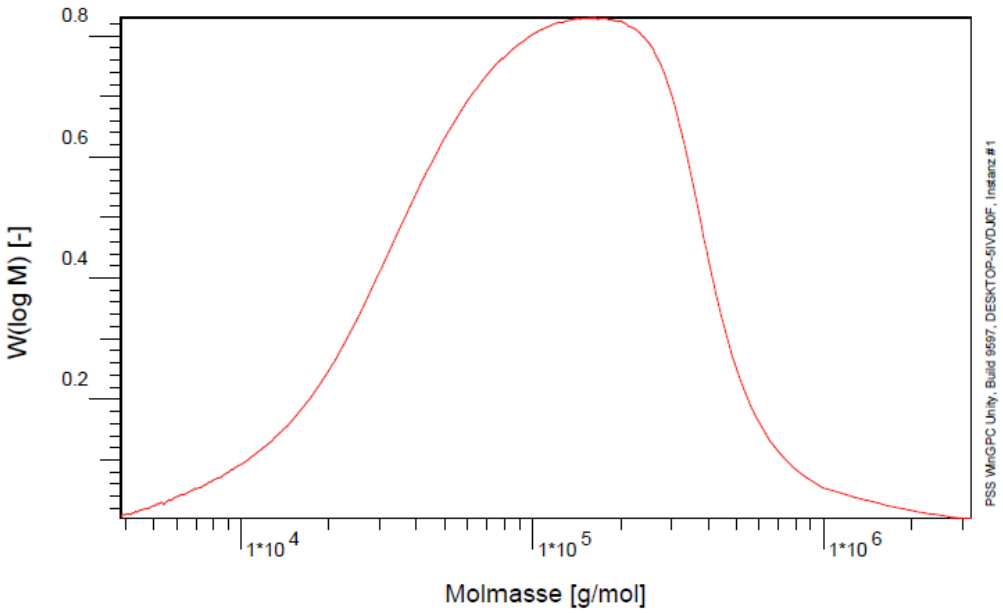

Figure S3: SEC graph of Poly(DMAPMA-co-MMES) (**3**)

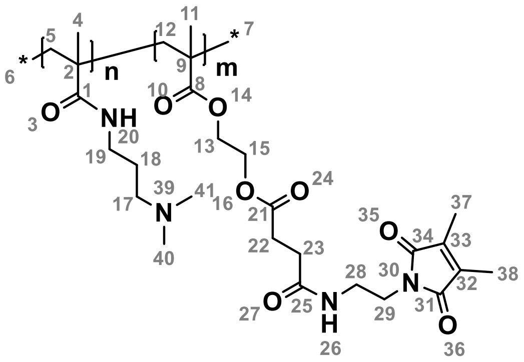

Figure S4: Molecular structure of Poly(DMAPMA-co-MME-DMMI) (**4**)

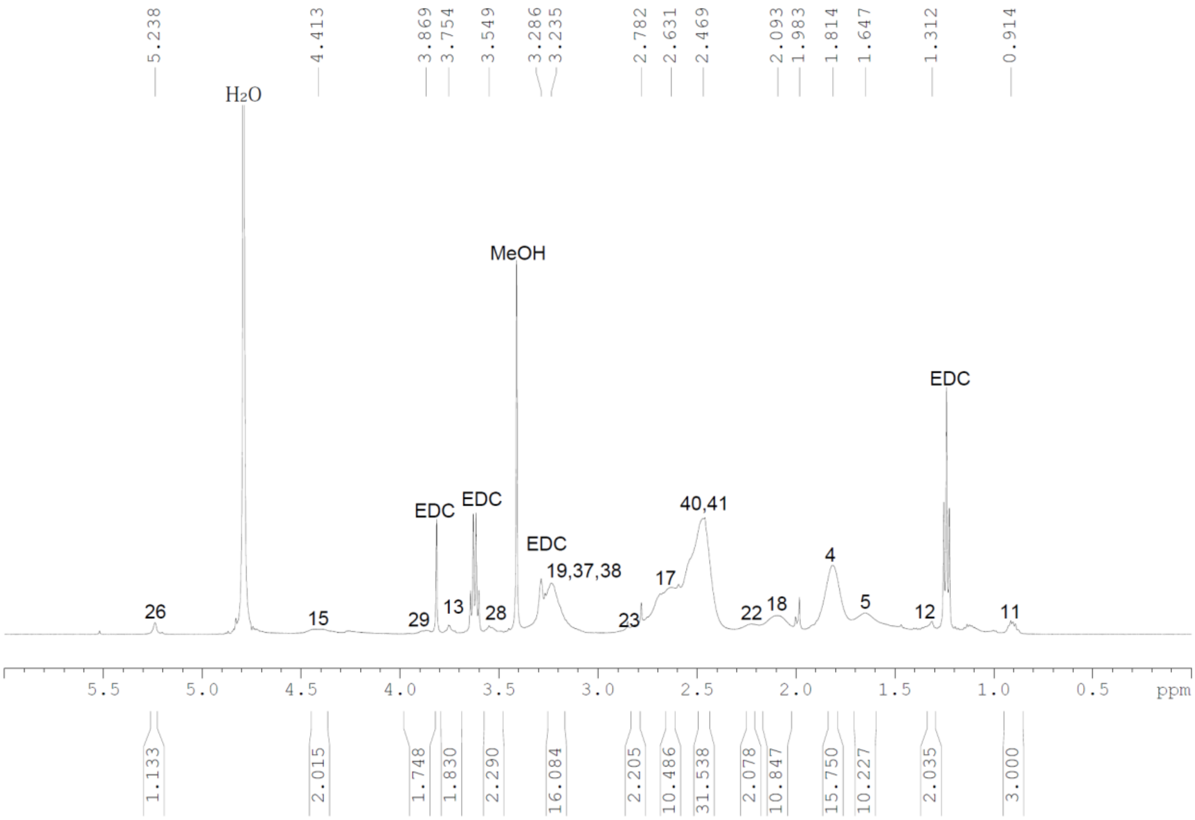

Figure S5: ^{1}H NMR spectrum of Poly(DMAPMA-co-MME-DMMI) (**4**) in D_2O

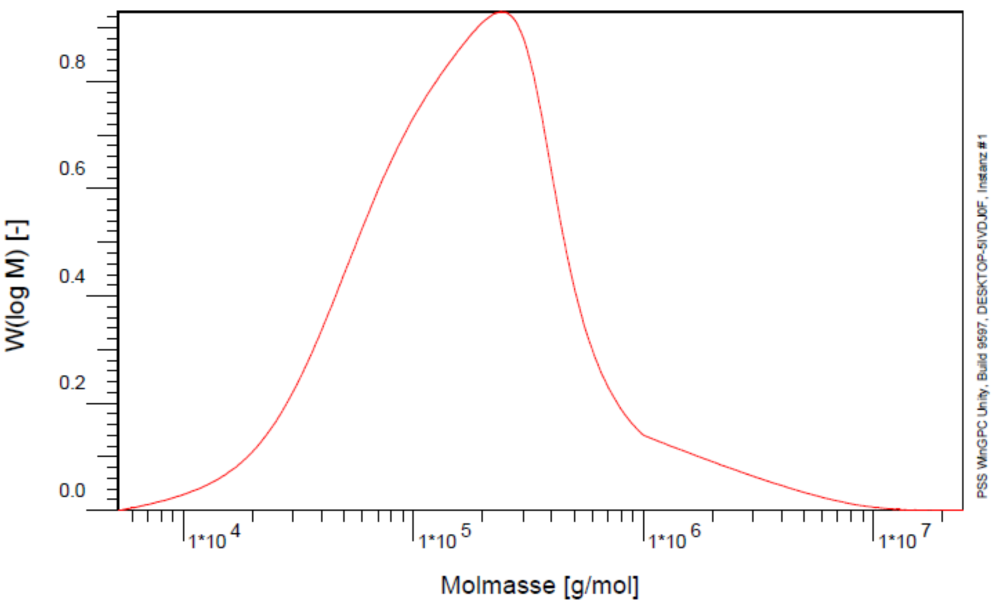

Figure S6: SEC graph of Poly(DMAPMA-co-MME-DMMI) (**4**)

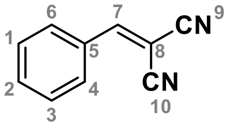

Figure S7: Molecular structure of 2-benzylidene malononitrile

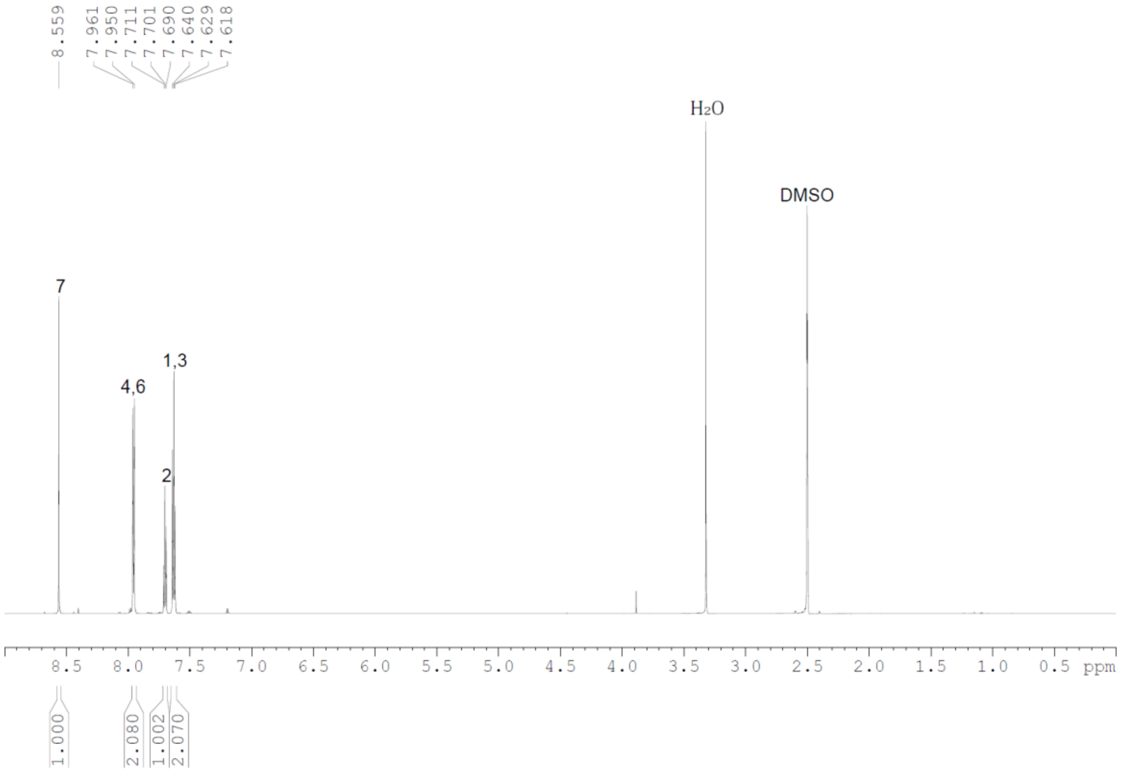

Figure S8: ^{1}H NMR spectrum of 2-benzylidene malononitrile DMSO-d_6

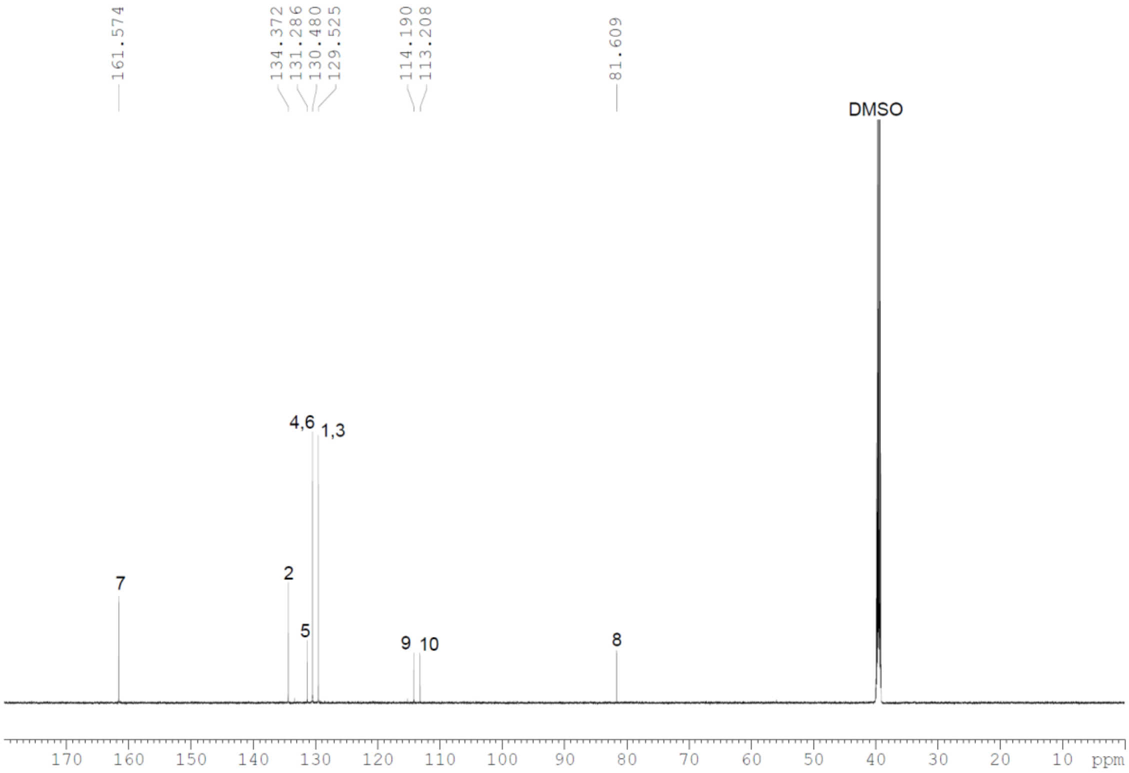

Figure S9: ^{13}C NMR spectrum of 2-benzylidene malononitrile DMSO-d_6

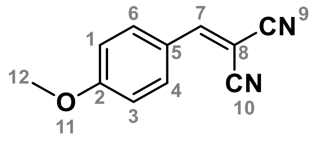

Figure S10: Molecular structure of 2-(4-methoxybenzylidene) malononitrile

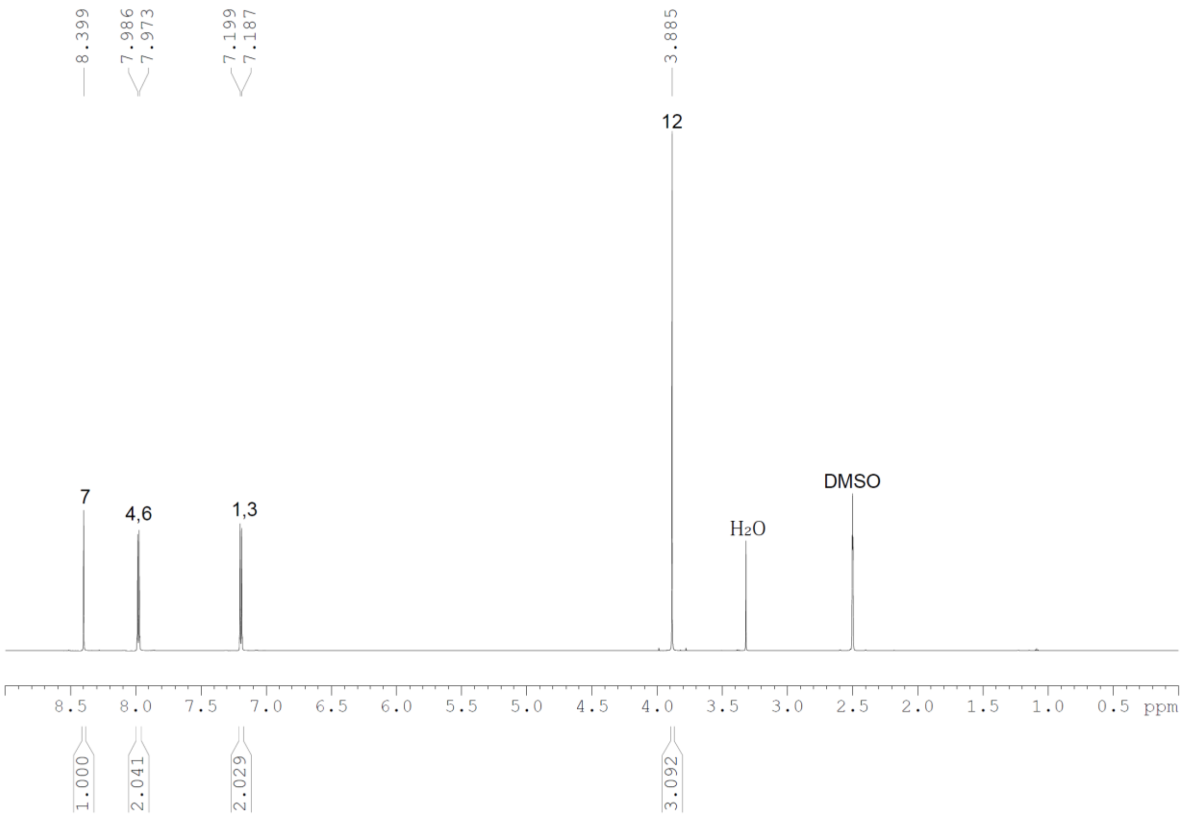

Figure S11: ^{1}H NMR spectrum of 2-(4-methoxybenzylidene) malononitrile DMSO-d_6

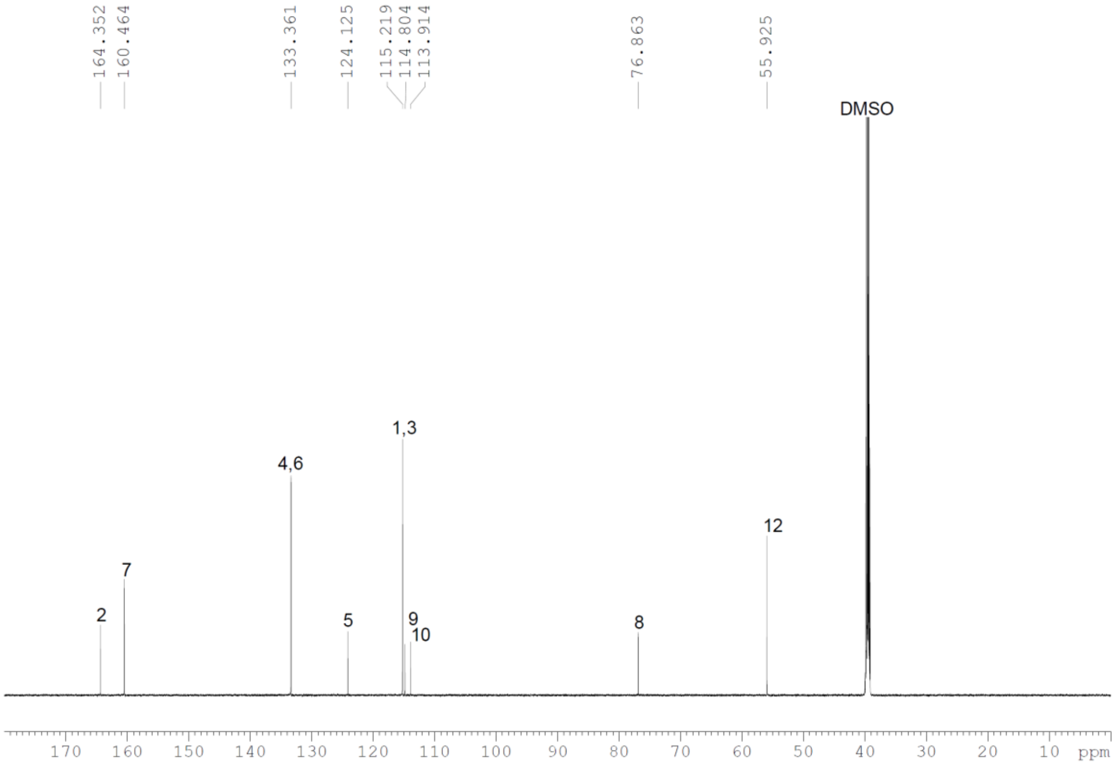

Figure S12: ^{13}C NMR spectrum of 2-(4-methoxybenzylidene) malononitrile DMSO-d_6

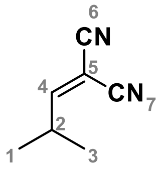

Figure S13: Molecular structure of 2-(2-methylpropylidene) malononitrile

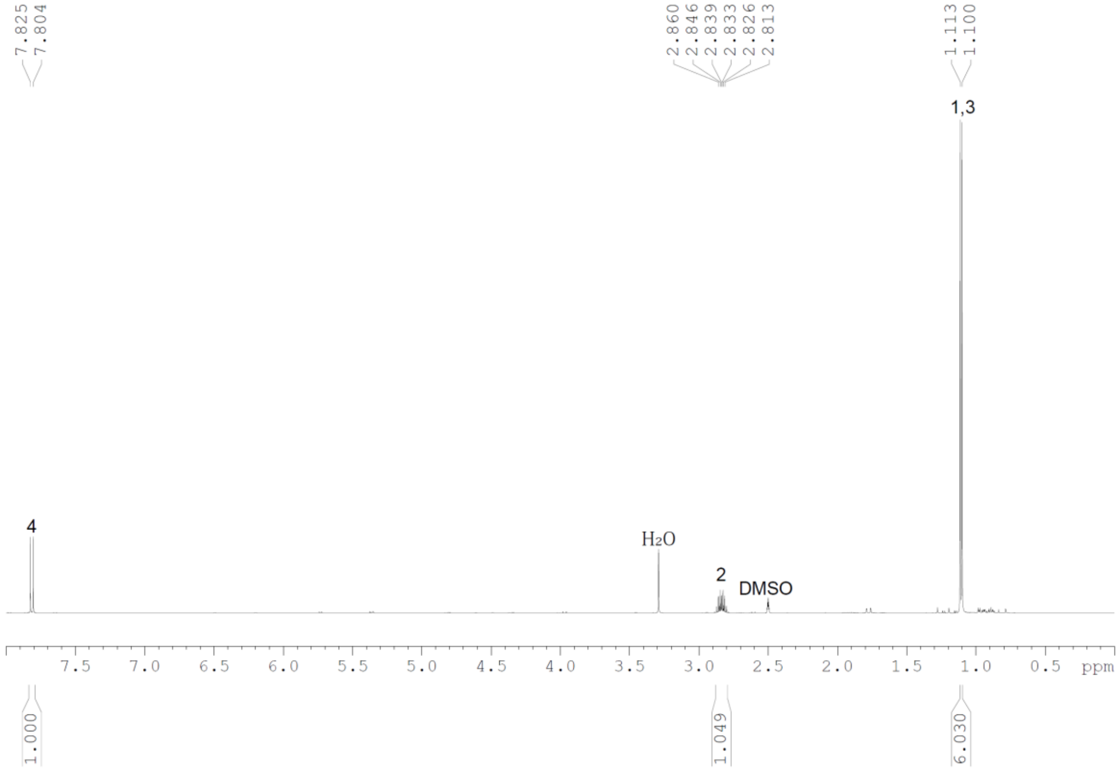

Figure S14: ^{1}H NMR spectrum of 2-(2-methylpropylidene) malononitrile DMSO-d_6

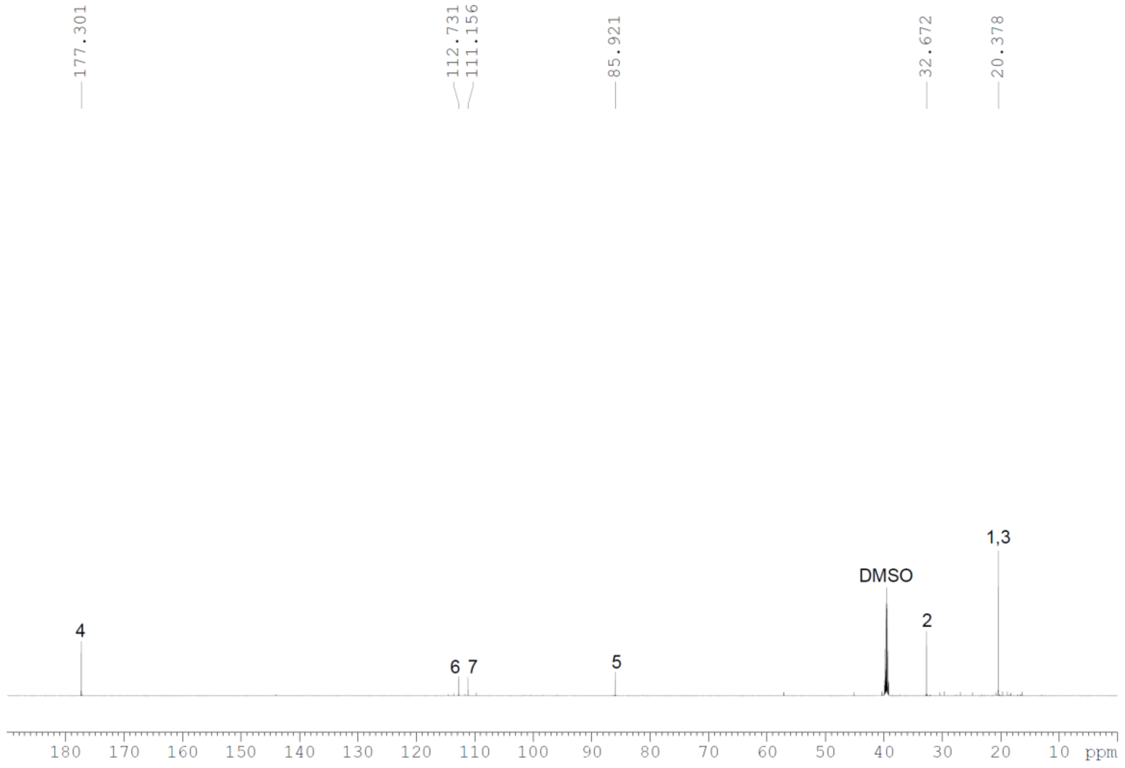

Figure S15: ^{13}C NMR spectrum of 2-(2-methylpropylidene) malononitrile DMSO-d_6

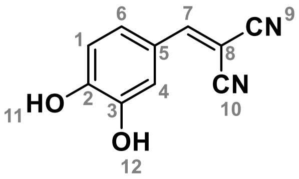

Figure S16: Molecular structure of 2-(3,4-dihydroxybenzylidene) malononitrile

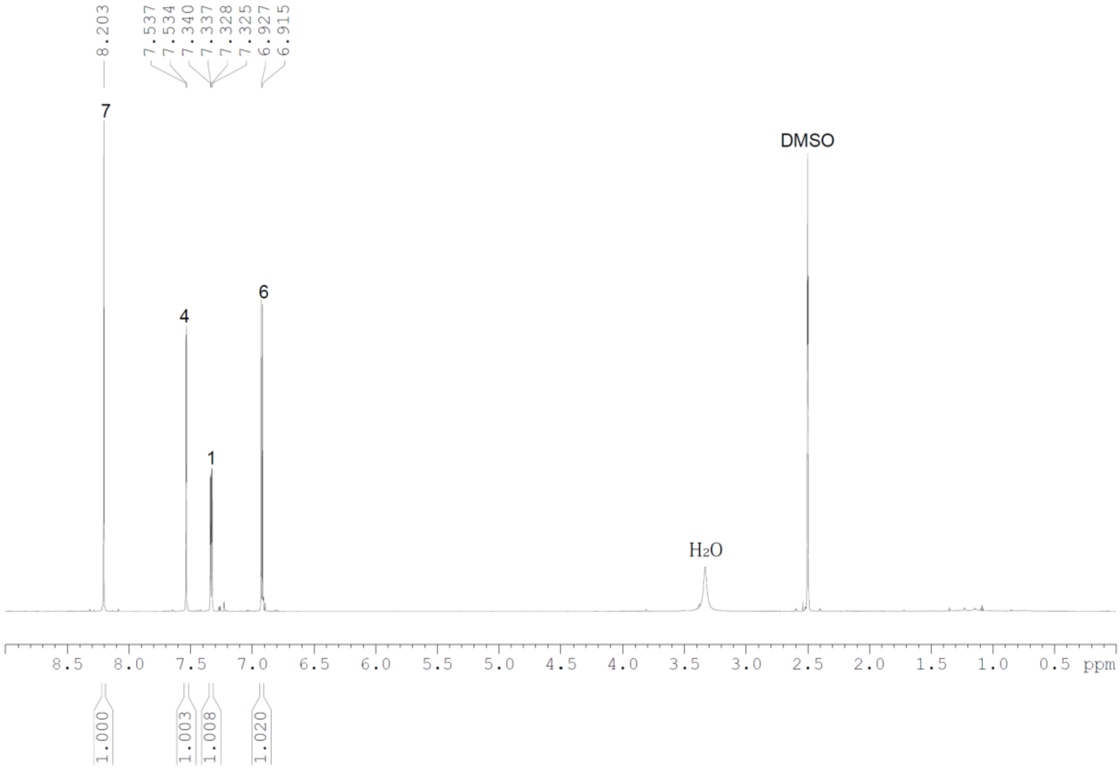

Figure S17: ^{1}H NMR spectrum of 2-(3,4-dihydroxybenzylidene) malononitrile in DMSO-d_6

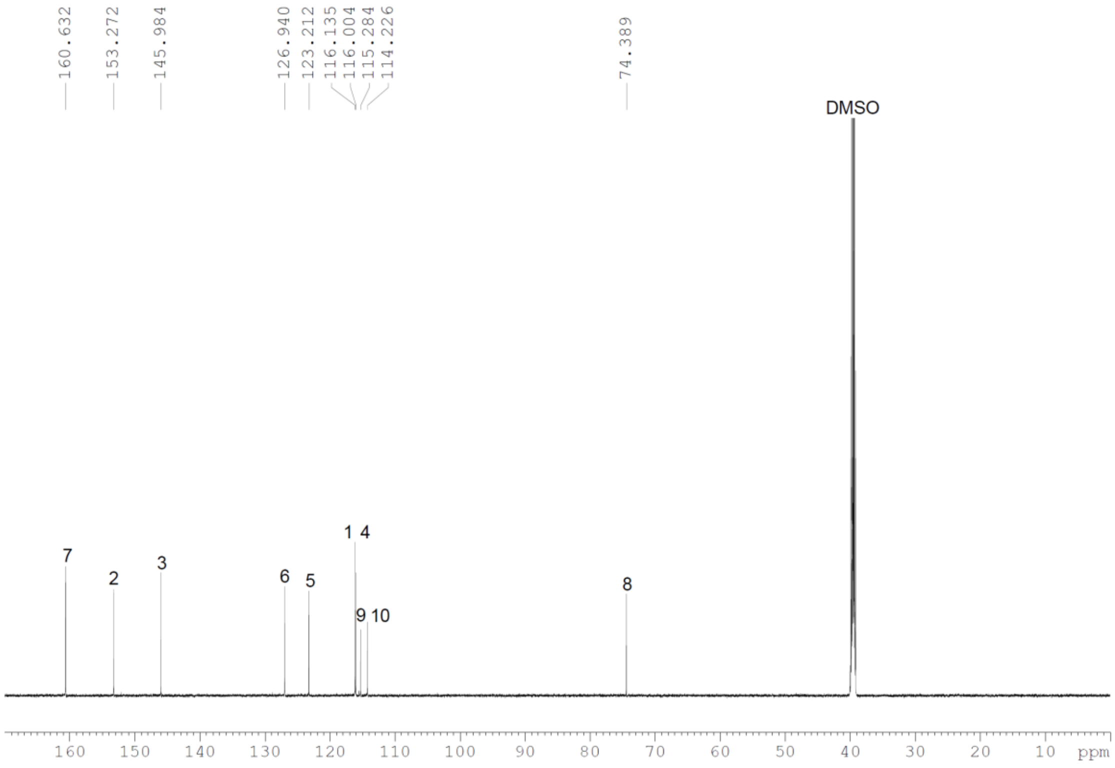

Figure S18: ^{13}C NMR spectrum of 2-(3,4-dihydroxybenzylidene) malononitrile in DMSO-d_6

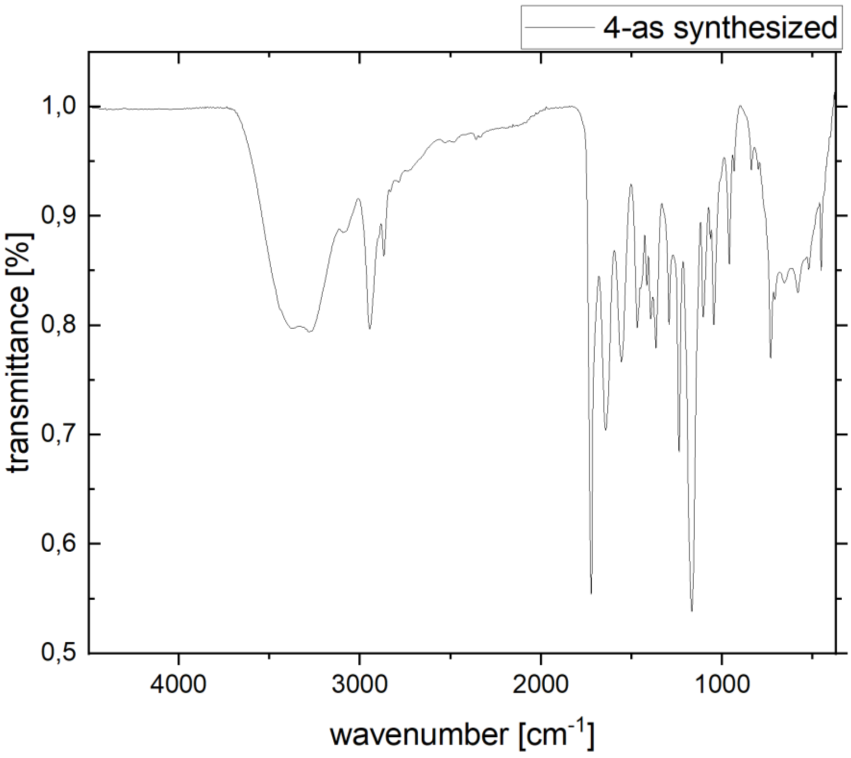

Figure S19: FTIR spectrum of Poly(DMAPMA-co-MMES) (**4**) as synthesized

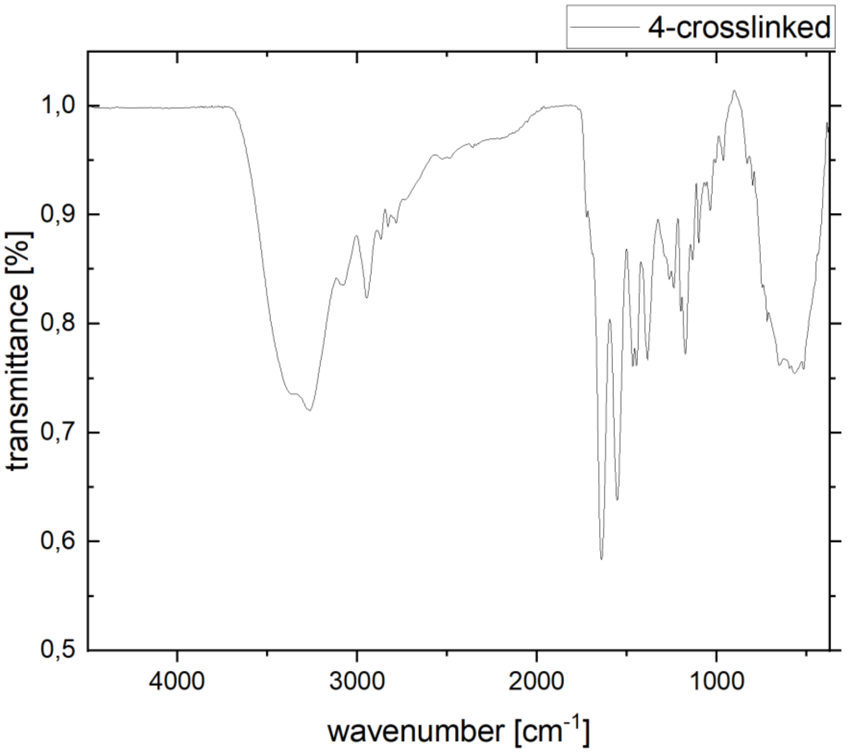

Figure S20: FTIR spectrum of Poly(DMAPMA-co-MMES) (**4**) after crosslinking

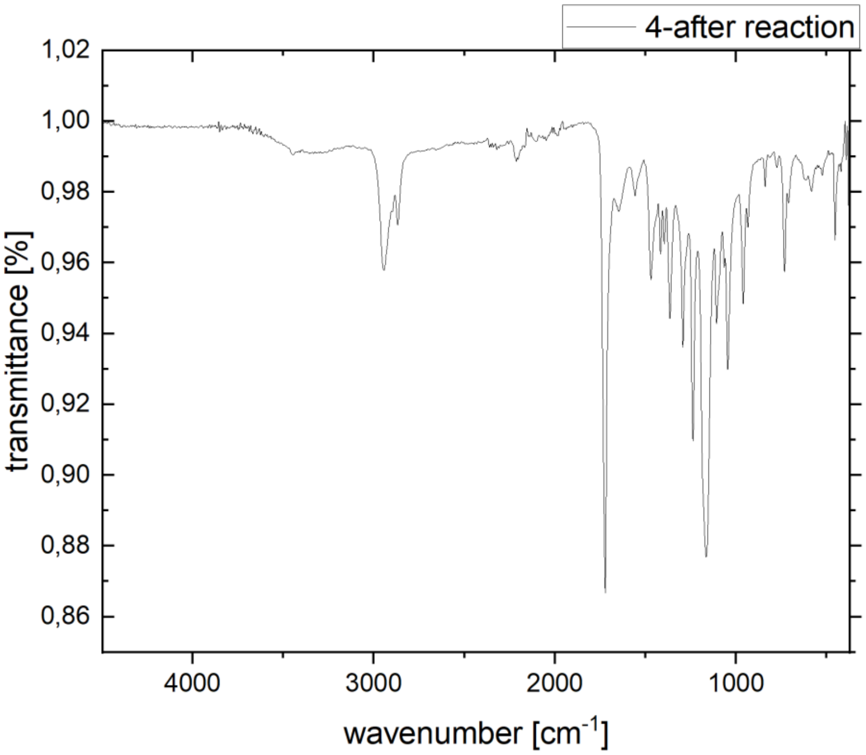

Figure S21: FTIR spectrum of Poly(DMAPMA-co-MMES) (**4**) after reaction of 3,4-dihydroxy benzaldehyde with malononitrile

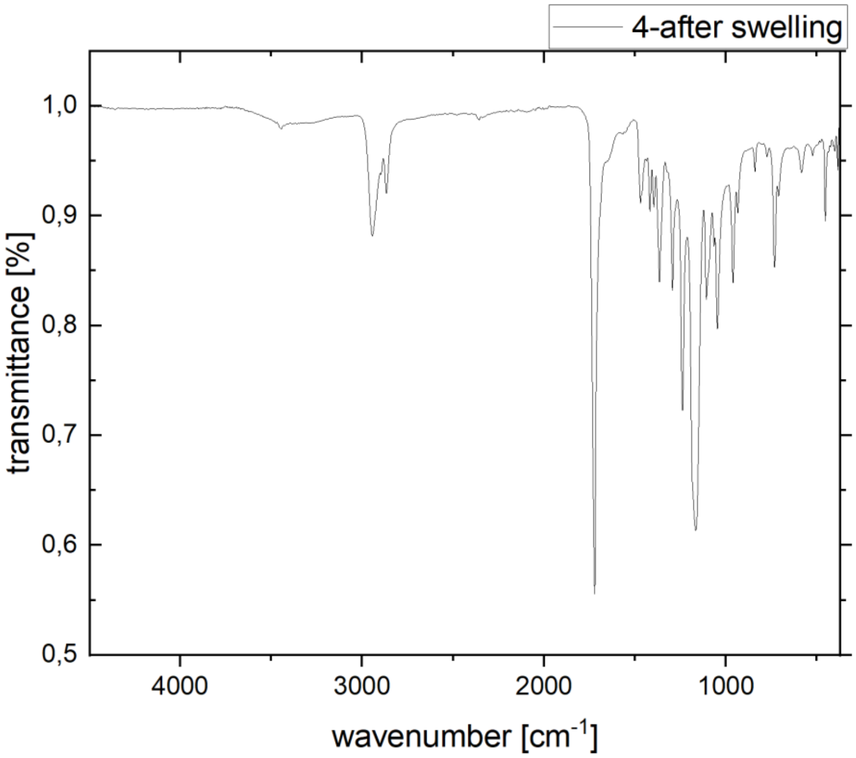

Figure S22: FTIR spectrum of Poly(DMAPMA-co-MMES) (**4**) after swelling in DMSO/IPrOH (1:1) for 2 h

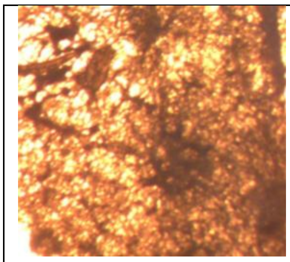	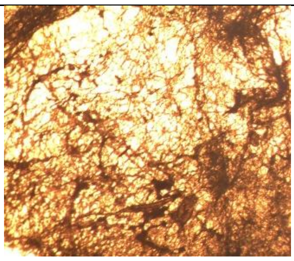	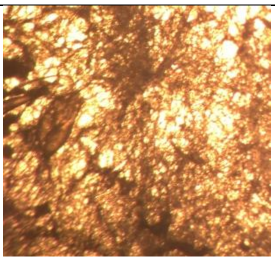	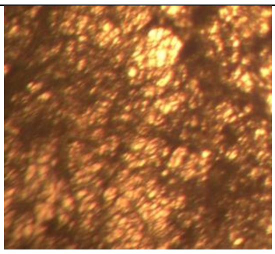
10x magnification	15x magnification	30x magnification	45x magnification

Figure S23: Light microscopic images of dry Poly(DMAPMA-co-MMES) (**4**) nanofiber mats with magnifications from 10x to 45x

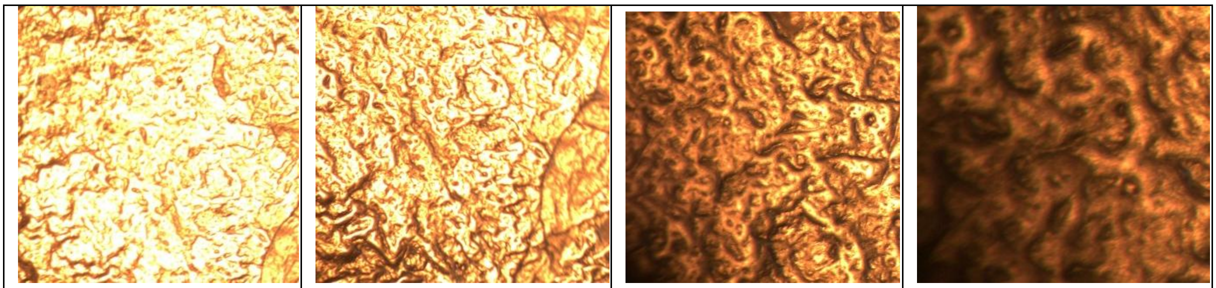

10x magnification	15x magnification	30x magnification	45x magnification

Figure S24: Light microscopic images of wet (DMSO/IPrOH 1:1) Poly(DMAPMA-co-MMES) (**4**) nanofiber mats with magnifications from 10x to 45x

Table S1: Percentages of solvent uptake in DMSO:IPrOH (1:1) of nanofiber mats after 1 h, 2 h, 3 h, 4h and 24 h

	$W_{M,1h}$ [%]	$W_{M,2h}$ [%]	$W_{M,3h}$ [%]	$W_{M,4h}$ [%]	$W_{M,24h}$ [%]
Poly(DMAPMA-co-MMES) (**4**)	170±33	329±63	481±160	575±203	511±82

Before	1h	2h	3h	4h	24h

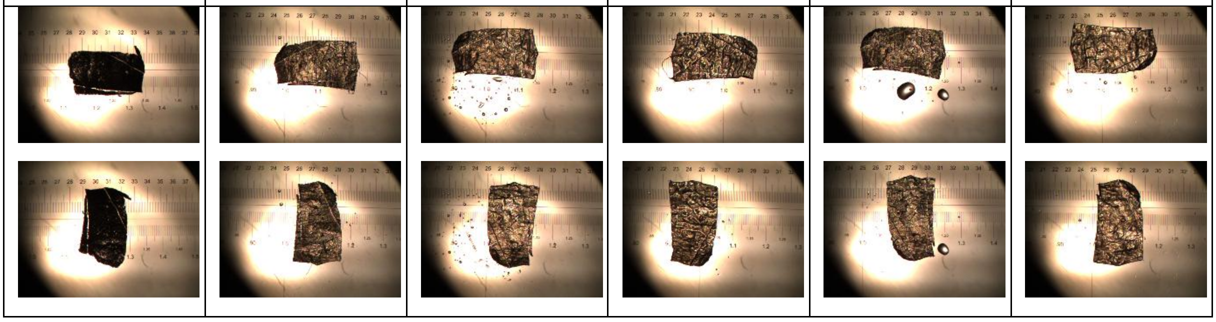

Figure S25: Light microscopic images of swollen Poly(DMAPMA-co-MMES) (**4**) nanofiber mats after 1 h, 2 h, 3 h, 4 h and 24 h in DMSO/IPrOH (1:1) (10x)

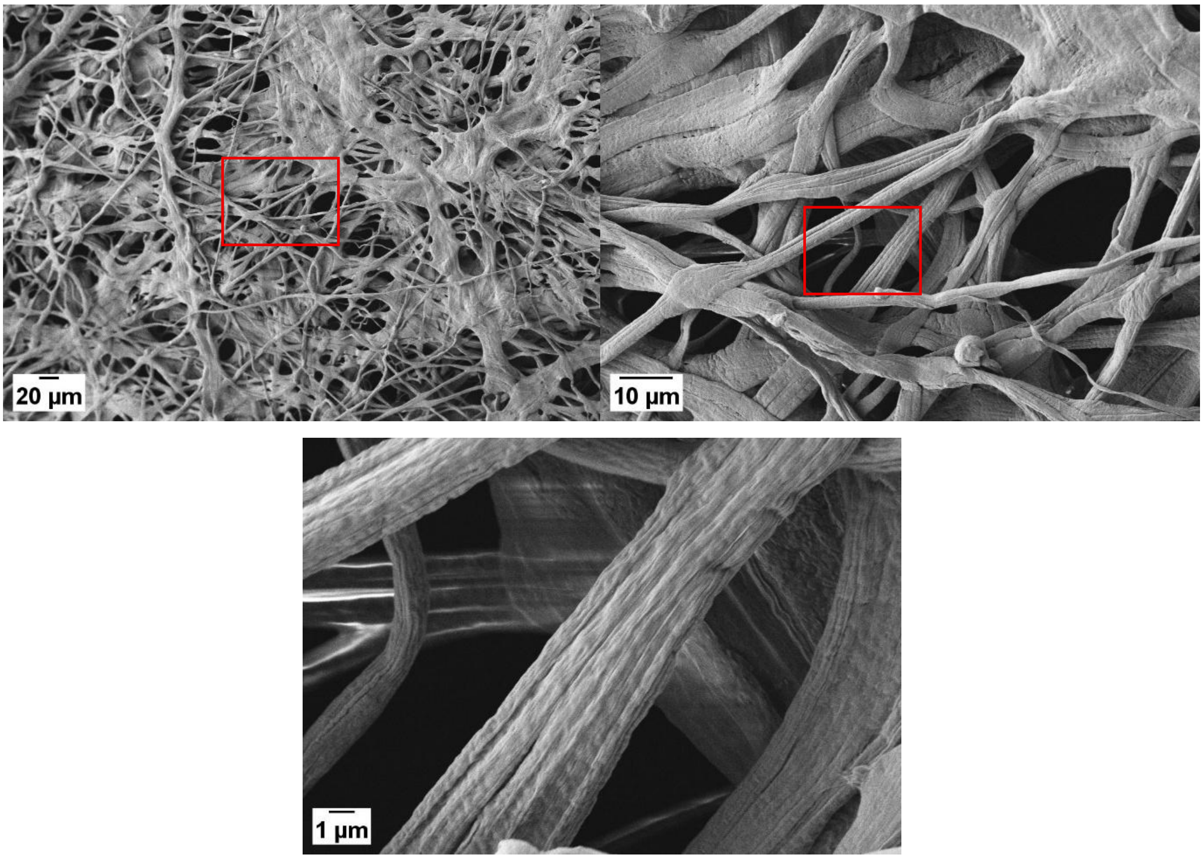

Figure S26-28: REM images of Poly(DMAPMA-co-MMES) (**4**) after crosslinking with magnification of 200x (Fig. S26), 1000x (Fig. S27) or 5000x (Fig. S28)

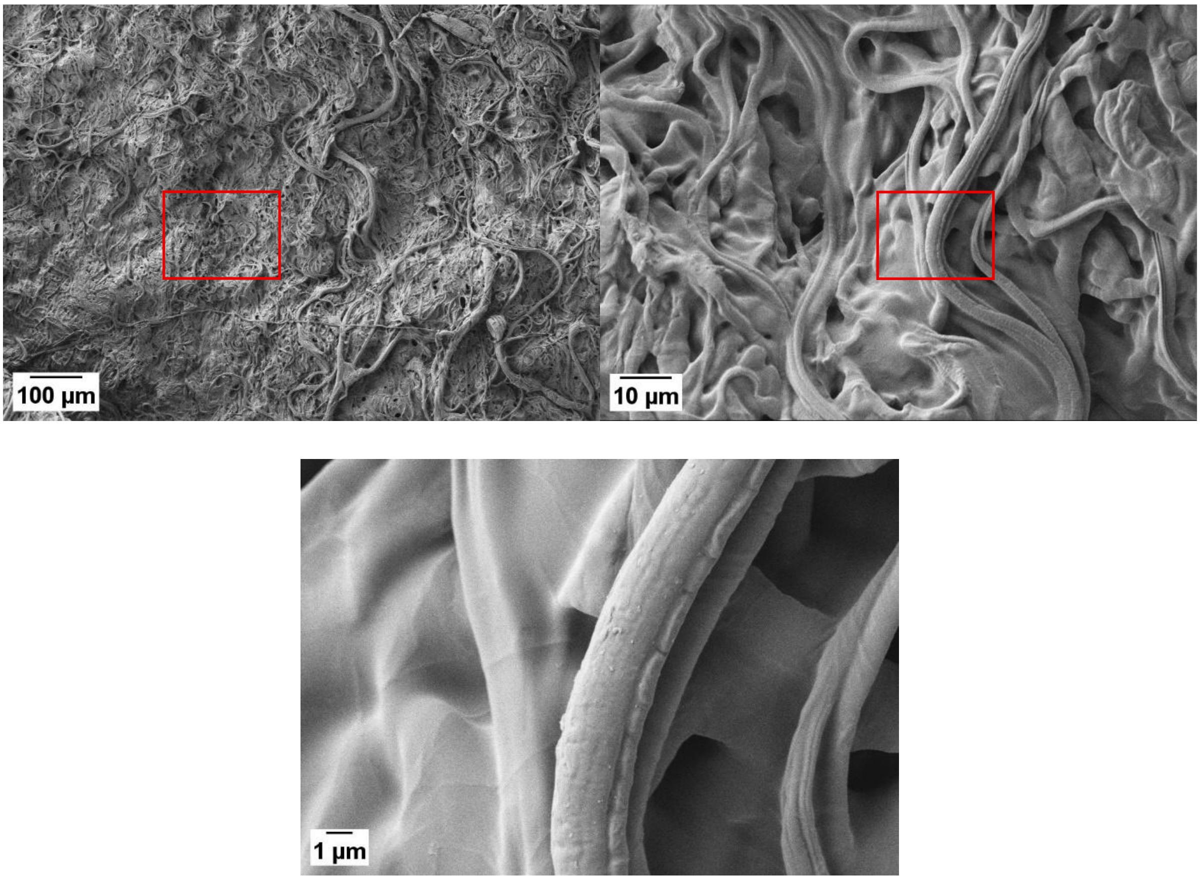

Figure S29-31: REM images of Poly(DMAPMA-co-MMES) (**4**) after 9 cycles of heterogeneous catalysis with magnification of 100x (Fig. S29), 1000x (Fig. S30) or 5000x (Fig. S31)

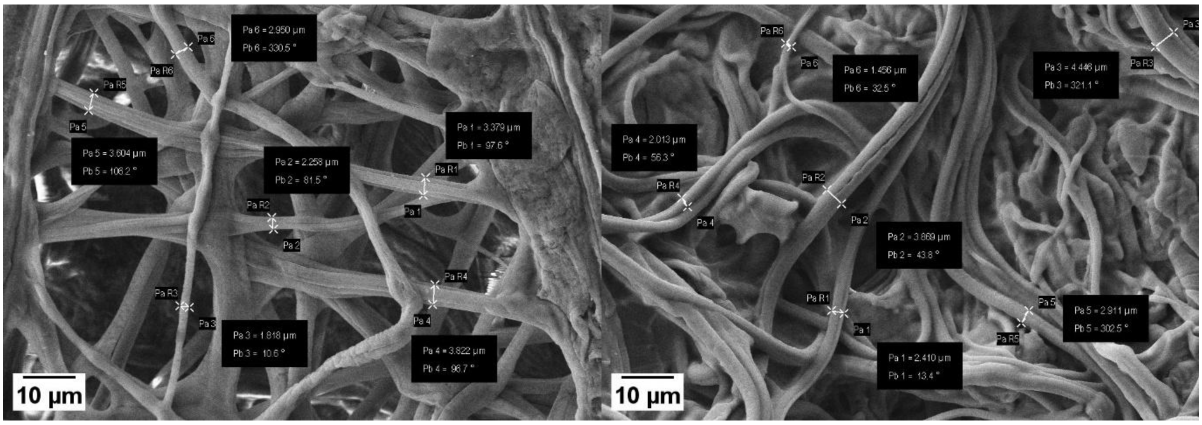

Figure S32-33: REM images of Poly(DMAPMA-co-MMES) (**4**) after crosslinking (Fig. S32) and after 9 cycles of heterogeneous catalysis (Fig. S33), which were used to determine the thickness of the fibers

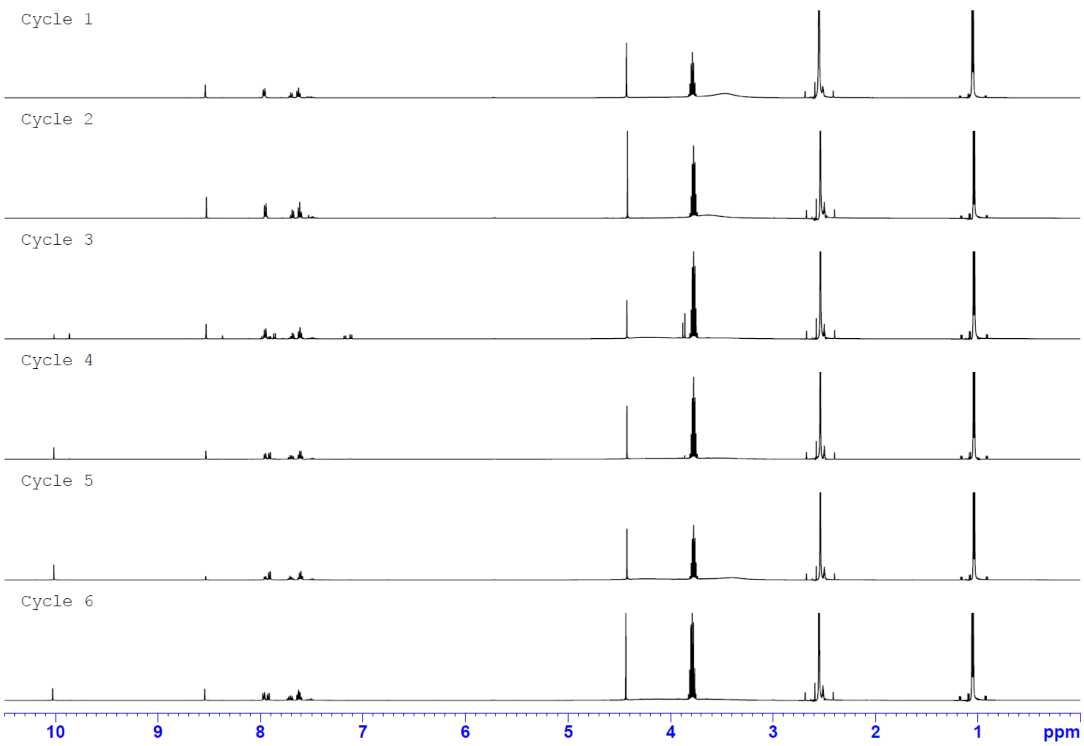

Figure S34: ^{1}H NMR spectra of the reaction mixture of benzaldehyde with malononitrile after 8 h of reaction time in six cycles with the same Poly(DMAPMA-co-MME-DMMI) (**4**) nanofiber mat in DMSO-d$_6$

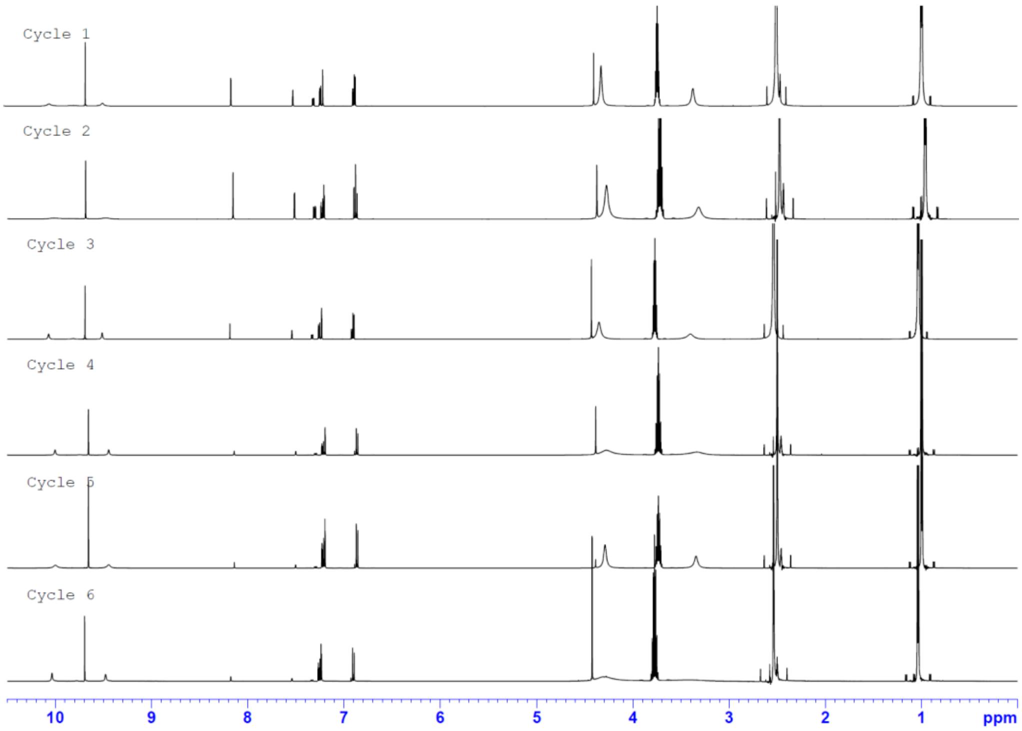

Figure S35: ^{1}H NMR spectra of the reaction mixture of 3,4-dihydroxy benzaldehyde with malononitrile after 8 h of reaction time in six cycles with the same Poly(DMAPMA-co-MME-DMMI) (**4**) nanofiber mat in DMSO-d$_6$

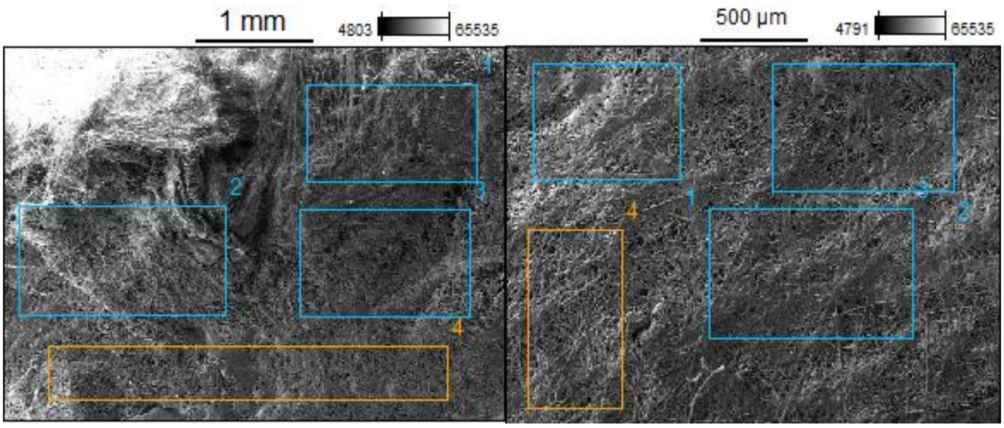

Figure S36: Sample segments 1-4 of EDX spectroscopy analysis (left, magnification 27x); Sample segments 5-8 of EDX spectroscopy analysis (right, magnification 50x)

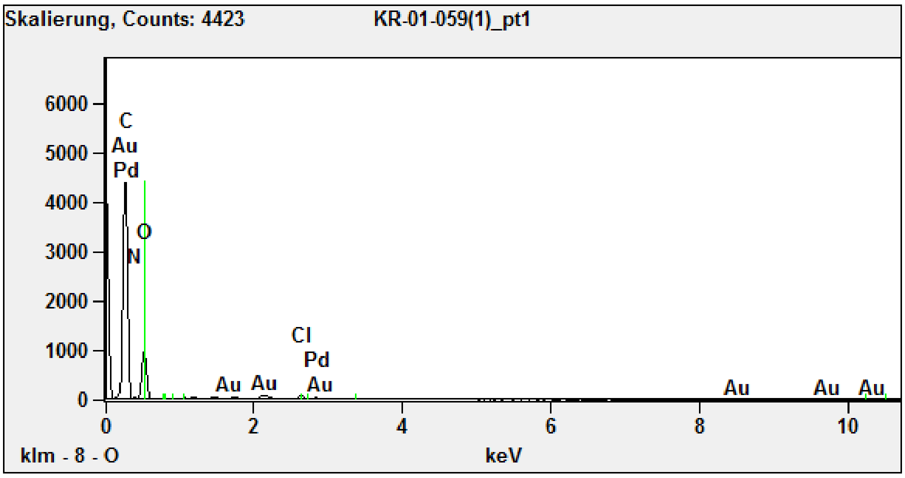

Figure S37: EDX spectrum of sample segment 1

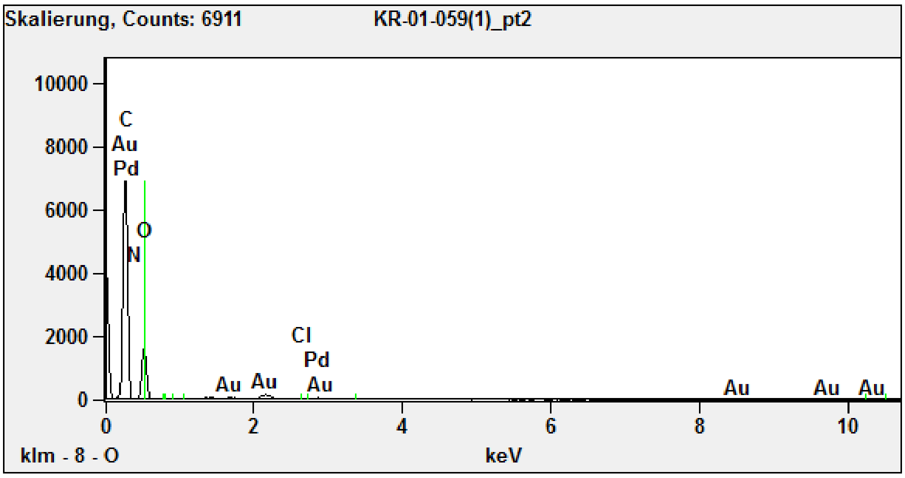

Figure S38: EDX spectrum of sample segment 2

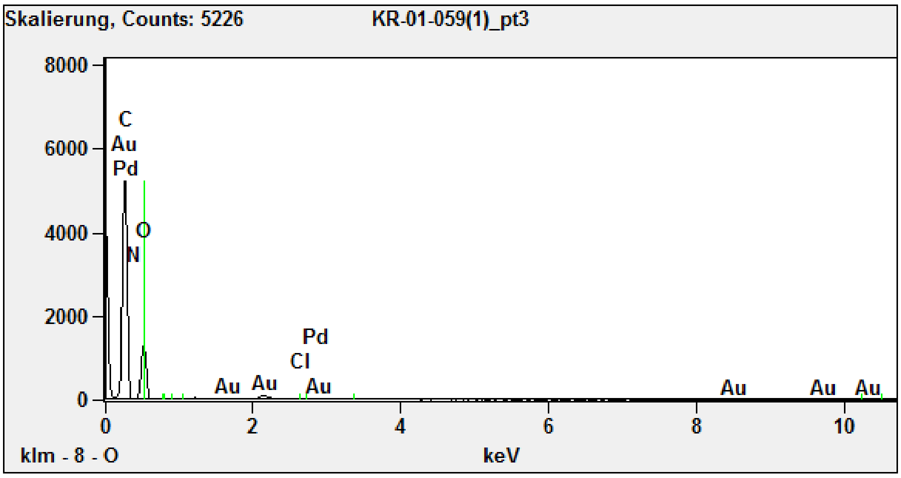

Figure S39: EDX spectrum of sample segment 3

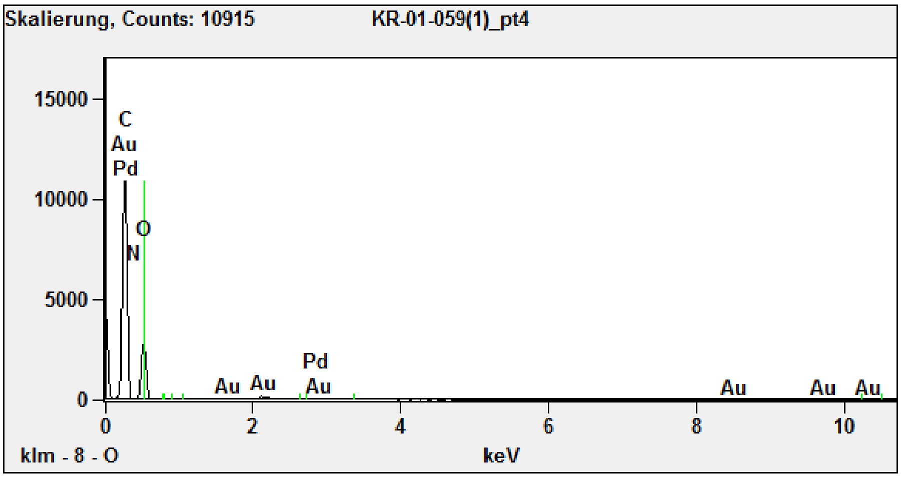

Figure S40: EDX spectrum of sample segment 4

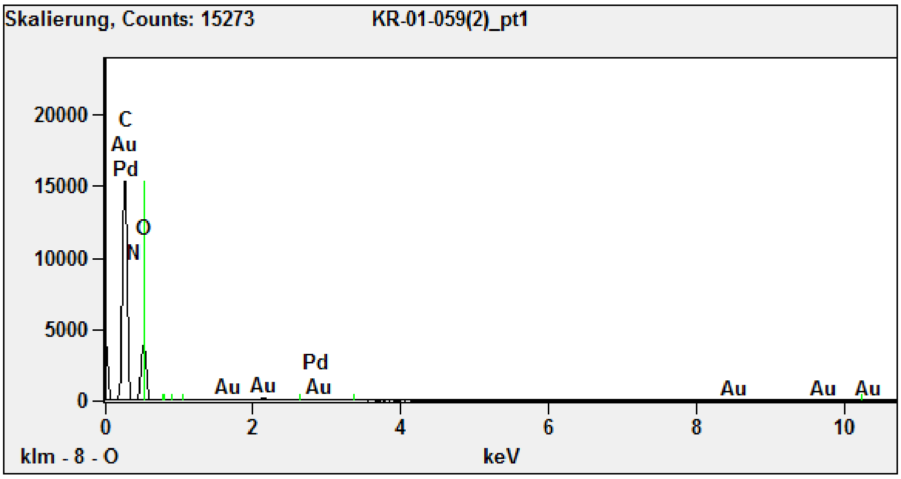

Figure S41: EDX spectrum of sample segment 5

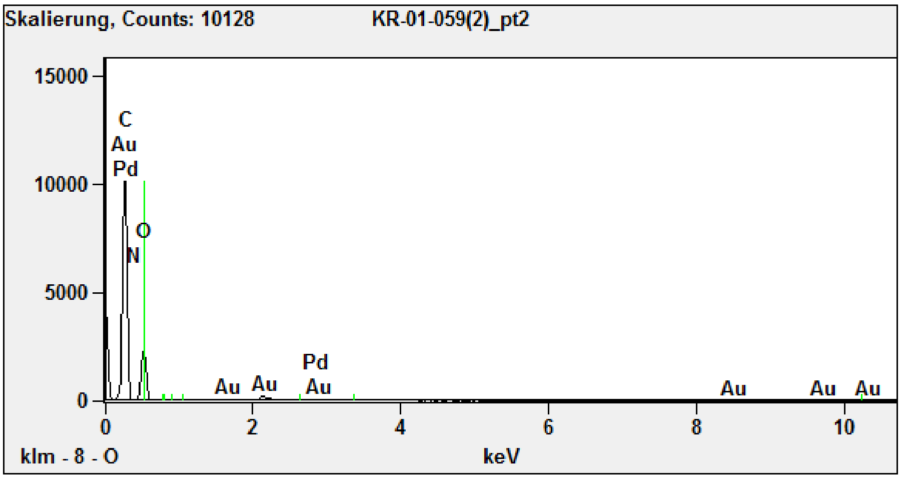

Figure S42: EDX spectrum of sample segment 6

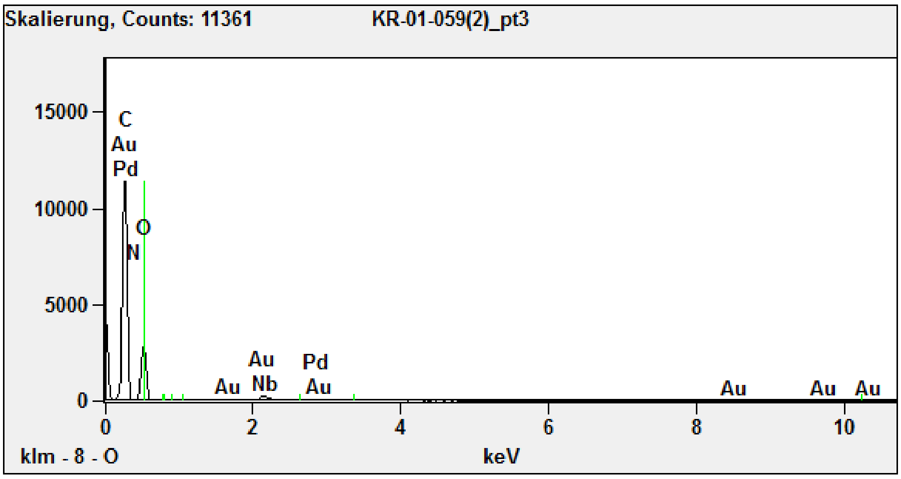

Figure S43: EDX spectrum of sample segment 7

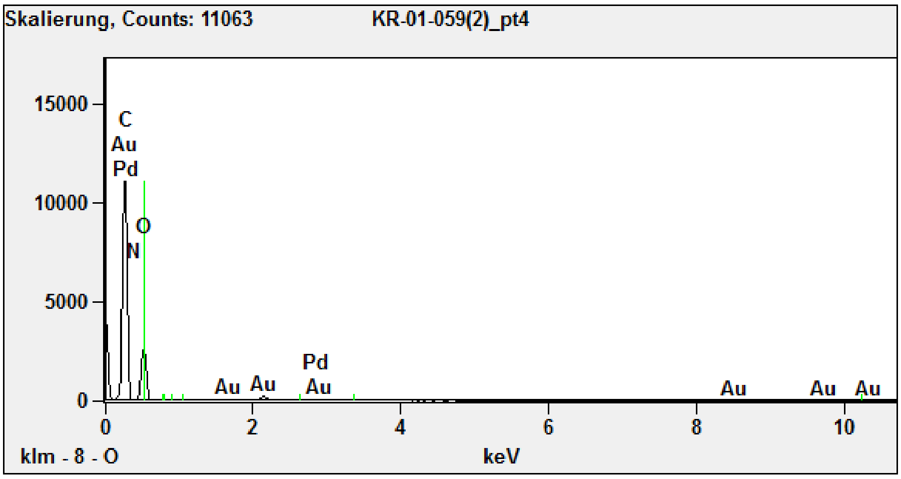

Figure S44: EDX spectrum of sample segment 8

Table S2: Wt.% and At.% results of EDX spectroscopy analysis of Poly(DMAPMA-co-MMES) (**4**)

Poly(DMAPMA-co-MMES) (**4**)	C-K	N-K	O-K	Cl-K	Pd-L	Au-M
Wt.%	44.29	6.30	41.41	0.47	2.10	5.44
Wt.% Error (±1σ)	0.34	1.17	0.59	0.10	0.34	0.34
At.%	54.35	6.64	38.14	0.18	0.29	0.41
At.% Error (±1σ)	0.42	1.23	0.55	0.03	0.05	0.02
